# Cancer stem-like properties and gefitinib resistance are dependent on purine synthetic metabolism mediated by the mitochondrial enzyme MTHFD2

**DOI:** 10.1038/s41388-018-0589-1

**Published:** 2018-12-07

**Authors:** Tatsunori Nishimura, Asuka Nakata, Xiaoxi Chen, Kurumi Nishi, Makiko Meguro-Horike, Soichiro Sasaki, Kenji Kita, Shin-ichi Horike, Kaori Saitoh, Keiko Kato, Kaori Igarashi, Takahiko Murayama, Susumu Kohno, Chiaki Takahashi, Naofumi Mukaida, Seiji Yano, Tomoyoshi Soga, Arinobu Tojo, Noriko Gotoh

**Affiliations:** 1 Division of Cancer Cell Biology, Cancer Research Institute, Kanazawa University, Kakuma-machi, Kanazawa city, 920-1192 Japan; 2 Division of Functional Genomics, Advanced Science Research Center, Kanazawa University, Takara-machi, Kanazawa city, 920-1192 Japan; 3 Division of Molecular Bioregulation, Cancer Research Institute, Kanazawa University, Kakuma-machi, Kanazawa city, 920-1192 Japan; 4 Division of Medical Oncology, Cancer Research Institute, Kanazawa University, 13-1, Takaramachi, Kanazawa city, 920-0934 Japan; 5 Institute for Advanced Biosciences, Keio University, 246-2, Minakami, Kakuganji, Tsuruoka city, Yamagata 997-0052 Japan; 6 Division of Molecular Therapy, Institute of Medical Science, University of Tokyo, 4-6-1 Shirokanedai, Minato-ku, Tokyo, 108-8639 Japan; 7 Division of Oncology and Molecular Biology, Cancer Research Institute, Kanazawa University, Kakuma-machi, Kanazawa city, 920-1192 Japan

**Keywords:** Cancer stem cells, Cancer metabolism

## Abstract

Tumor recurrence is attributable to cancer stem-like cells (CSCs), the metabolic mechanisms of which currently remain obscure. Here, we uncovered the critical role of folate-mediated one-carbon (1C) metabolism involving mitochondrial methylenetetrahydrofolate dehydrogenase 2 (MTHFD2) and its downstream purine synthesis pathway. *MTHFD2* knockdown greatly reduced tumorigenesis and stem-like properties, which were associated with purine nucleotide deficiency, and caused marked accumulation of 5-aminoimidazole carboxamide ribonucleotide (AICAR)—the final intermediate of the purine synthesis pathway. Lung cancer cells with acquired resistance to the targeted drug gefitinib, caused by elevated expression of components of the β-catenin pathway, exhibited increased stem-like properties and enhanced expression of MTHFD2. *MTHFD2* knockdown or treatment with AICAR reduced the stem-like properties and restored gefitinib sensitivity in these gefitinib-resistant cancer cells. Moreover, overexpression of MTHFD2 in gefitinib-sensitive lung cancer cells conferred resistance to gefitinib. Thus, MTHFD2-mediated mitochondrial 1C metabolism appears critical for cancer stem-like properties and resistance to drugs including gefitinib through consumption of AICAR, leading to depletion of the intracellular pool of AICAR. Because CSCs are dependent on MTHFD2, therapies targeting MTHFD2 may eradicate tumors and prevent recurrence.

## Introduction

An increase in the incidence and mortality rate of cancer has been noted worldwide, especially for lung cancer [1]. Many patients with adenocarcinoma, a major subtype of lung cancer, harbor mutations in the epidermal growth factor receptor (EGFR) in their cancer tissues and initially show a good response to molecular targeted drugs such as gefitinib, which inhibits the EGFR tyrosine kinase [2, 3]. However, recurrence inevitably occurs within a few years owing to acquired drug resistance, leading to poor prognosis [4, 5]. Despite the development of several molecular targeted drugs, the problems of recurrence and acquired drug resistance remain unsolved.

Emerging evidence suggests that cancer stem-like cells (CSCs) are responsible for tumor recurrence and drug resistance [6]. Tumor tissues are composed of heterogeneous cell types, including CSCs and their differentiated progeny, as observed in normal tissues derived from tissue-specific stem cells [7–9]. Most cancer therapies such as conventional chemotherapy, radiotherapy, and molecular targeted drugs target rapidly proliferating differentiated cancer cells, but not CSCs. Cancer cells with stem-like properties are thought to continue to survive after these treatments, and a fraction of these cells exhibit the ability to regrow after years, causing recurrence. Thus, targeted therapies against CSCs together with their progeny cancer cells are important for tumor eradication; however, these are still unmet medical needs. To establish such therapies, the molecular mechanisms that determine how CSCs are maintained in cancer tissues need to be elucidated. We and other researchers have previously shown that growth factors appear to maintain CSCs in cancer tissues [10–12]. Growth factors regulate many aspects of cancer biology, including metabolism in cancer cells. Although various metabolic pathways are believed to be activated in cancer cells, the specific metabolic mechanisms in CSCs remain largely unclear.

**Fig. 1:**
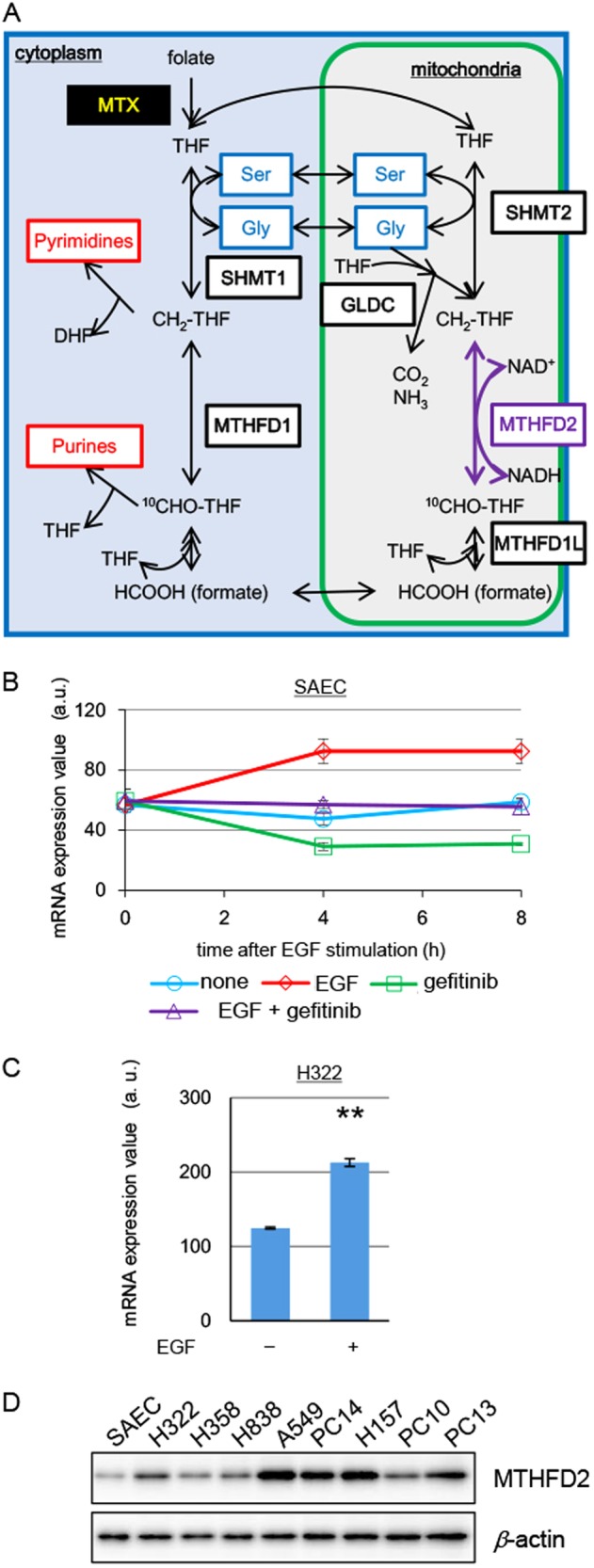
A map of folate-mediated 1C metabolism and expression of *MTHFD2* in lung cancer tissues and EGF-stimulated cells. **a** Folate-mediated 1C metabolism. The reaction catalyzed by MTHFD2 is depicted with purple arrows. Major amino acids and enzymes involved in 1C metabolism are written in blue and black characters in boxes, respectively. MTX methotrexate. **b** Time-dependent *MTHFD2* mRNA levels in SAECs stimulated with or without EGF (100 ng/ml) in the presence or absence of gefitinib (1 μM) were measured by quantitative RT-PCR. The data are represented as mean ± SD, *N* = 3. **c** *MTHFD2* mRNA levels in H322 cells treated with or without EGF (100 ng/ml) for 8 h were measured by quantitative RT-PCR. Experiments were performed three times and the representative results were presented. The data are represented as mean ± SD, *N* = 3. Statistical significance is calculated with a two-tailed unpaired *t*-test; **p < 0.01. **d** Western blot for MTHFD2 in lung cancer cell lines and normal lung epithelial cells (SAECs). *β*-Actin was used as loading control

One-carbon (1C) metabolism incorporates carbons as building blocks of purine and pyrimidine and is important for DNA replication, RNA transcription, and cell proliferation [13–15] (Fig. 1a). These carbons are transferred from serine or glycine, and folate-derived tetrahydrofolate (THF) serves as a cofactor to produce CH_2_–THF. 1 C derived from CH_2_–THF is incorporated into pyrimidines, and two parallel reactions take place involving CH_2_–THF to produce ^10^CHO–THF in the mitochondria and cytoplasm. In the mitochondria, methylenetetrahydrofolate dehydrogenase 2 (MTHFD2) uses CH_2_–THF and NAD^+^ as substrates to catalyze the production of ^10^CHO–THF and NADH. Subsequently, ^10^CHO–THF is used to produce formate that can penetrate through the mitochondrial membrane and be transported to the cytoplasm to serve as a substrate for ^10^CHO–THF production. 1 C derived from ^10^CHO–THF is incorporated into purines. Although these enzymes are able to catalyze reactions in both directions, these reactions likely occur in the above-mentioned direction, because CH_2_–THF and ^10^CHO–THF are used to produce pyrimidines and purines, respectively [16, 17]. Methotrexate (MTX), a conventional anticancer drug, blocks 1C metabolism both in the cytoplasm and mitochondria, causing strong side effects (Fig. 1a) [13]. On the other hand, MTHFD2 and several other enzymes in the mitochondria are expressed during embryonic stages, but not after birth, in most normal tissues. MTHFD2-deficient mice show embryonic lethality due to defects in growth of fetal liver progenitor cells [18]. This indicates that MTHFD2 is necessary for growth of embryonic cells. In contrast, MTHFD2 may be dispensable for cell growth in normal adult tissues as suggested by its low expression. Expression of these mitochondrial enzymes is increased in cancer cells, and thus, 1C metabolism occurring in the mitochondria has been recognized as an important potential drug target in cancer with possible minimal side effects [19–24]. However, the role of mitochondrial 1C metabolism in lung cancer is still unclear. Furthermore, whether mitochondrial 1C metabolism plays any role in drug-resistant CSCs is still largely unknown.

The purine intermediate 5-aminoimidazole carboxamide ribonucleotide (AICAR) has multiple functions, including inhibition of cell growth and activation of AMP-activating protein kinase (AMPK) [25, 26]. Activated AMPK phosphorylates downstream substrates, leading to an increase in the rate of catabolic (ATP-producing) pathways and a decrease in the rate of anabolic (ATP-utilizing) pathways [27, 28]. However, the role of AICAR in drug resistance and cancer stem-like properties is still largely unknown.

Several mechanisms determine how lung adenocarcinoma patients acquire resistance to gefitinib, including the appearance of additional *EGFR* mutations that lead to decreased affinity for gefitinib and amplification of the *MET* receptor tyrosine kinase. T790M (threonine 790 is replaced by methionine) in *EGFR* is the most common mutation, whereas *MET* is amplified in other cases [29]. We and other researchers have shown that elevated expression of components of the β-catenin pathway is a resistance mechanism against gefitinib, even if there are neither additional mutations in *EGFR* nor *MET* amplification [30, 31]. Many drugs targeting molecules of the β-catenin pathway have been developed; however, only a few of these are clinically useful, as targeting the β-catenin pathway, which plays important roles in many normal cells (e.g., intestinal stem cells), causes major side effects [32]. In this study, we show that acquired gefitinib resistance in cancer cells caused by elevated expression of the components of the β-catenin pathway was associated with stem-like properties, indicating that prolonged gefitinib treatment does not eliminate CSCs, and instead may enrich them.

We previously showed that the EGF-regulated 139-gene signature can accurately determine the prognosis of adenocarcinoma patients [33]. In other words, the expression levels of these genes correlate well with the grade of malignancy. In this study, we examined the possibility of previously unrecognized critical tumorigenic genes among the 139 genes that may be novel molecular targets. We identified *MTHFD2* as a molecular target for lung cancer and showed that MTHFD2-dependent 1C metabolism is a common critical mechanism for the growth of CSCs and drug-resistant cells, as well as cancer cells in which MTHFD2 is expressed at high levels. Moreover, MTHFD2 plays important roles in drug resistance and cancer stem-like properties by depleting the AICAR intracellular pool.

## Results

### MTHFD2 is a druggable molecular target in lung cancer

Activation of the EGFR signaling pathway plays a crucial role in many aspects of cancer biology [34]. We have proposed that the EGF-regulated 139-gene signature is useful for the prognosis of patients with lung adenocarcinoma [33]. We chose candidate molecular targets from the 139 genes according to the following selection processes: Among the EGF-regulated 139 genes, we analyzed expression levels of each gene in 226 lung adenocarcinoma tissues in the National Cancer Center (NCC) cohort [35]. We used the median values as the cutoff values to obtain Kaplan–Meier relapse-free survival curves. We selected 42 genes of which their high expression levels were significantly associated with a worsened prognosis by log-rank test (*p* < 0.05). We analyzed other cohorts using Prognoscan (http://www.abren.net/PrognoScan/) and confirmed that high expression levels of these genes were associated with a worsened prognosis. Among the 42 genes, we analyzed the Oncomine database (https://www.oncomine.org/resource/login.html) and selected 13 genes of which expression levels were significantly higher ( > 2-fold) in lung adenocarcinoma tissues as compared to normal tissues in more than one cohort. Some are well-known molecular targets, including *MMP1*, *MMP12*, and *VEGFA*. We selected MTHFD2 as a novel target and found potential binding pockets for small compounds with the PockDock software [36] (Supplementary Figure 1A-D).

We examined the expression of *MTHFD2* in normal lung epithelial cells (small airway epithelial cells (SAECs)). *MTHFD2* expression was induced by EGF stimulation and was nearly undetectable after pretreatment with gefitinib (Fig. 1b). EGF treatment induced *MTHFD2* expression in H322, but little, if any, in A549 lung cancer cells (Fig. 1c and Supplementary Figure 2A). Collectively, these results confirmed that *MTHFD2* was upregulated upon EGF stimulation in some normal and cancer cells.

### MTHFD2 plays important roles in cell growth and tumorigenesis in lung cancer cells

Western blotting with samples of SAECs and various lung cancer cell lines showed that MTHFD2 was expressed at varying levels among the lung cancer cell lines (Fig. 1d). In contrast, very low expression of MTHFD2 was detected in SAECs. We examined the role of MTHFD2 in A549 and H322 lung cancer cells that show high-level and moderate expression of MTHFD2, respectively. Previously, it was reported that MTHFD2 is localized not only in the mitochondria, but also in the nucleus of HCT-116 and HeLa cells [37]. Consistent with this, MTHFD2 was localized in the mitochondria and nucleus of both H322 and A549 cells (Supplementary Figure 2B).

**Fig. 2:**
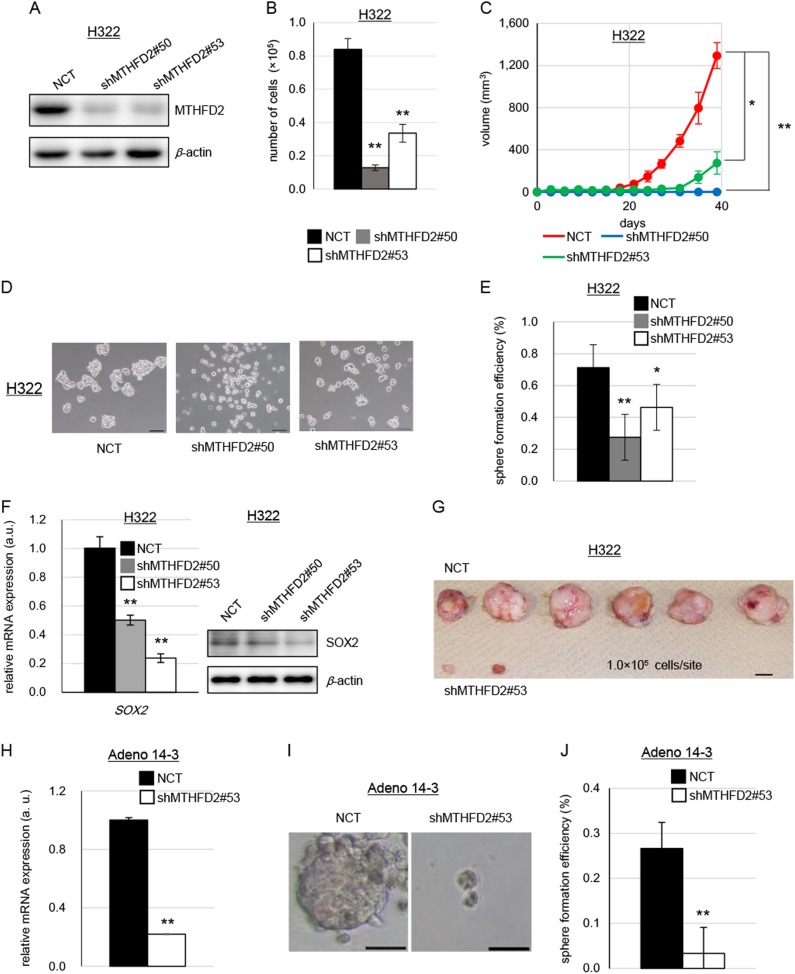
Reduced cellular growth and cancer stem-like properties following knockdown of *MTHFD2*. **a** Western blot for MTHFD2 in H322 cells in which *MTHFD2* was knocked down with two different shRNAs. NCT negative control; shMTHFD2#50 and shMTHFD2#53, *MTHFD2* knockdown. **b** Cell proliferation assay of H322 cells *in vitro*. Experiments were performed three times and the representative results were presented. The data are represented as mean ± SD, *N* = 3. **c** Tumor growth assay of H322 cells in vivo. The data are represented as mean ± SEM, *N* = 4. **d** Bright field images of tumor spheroids of *MTHFD2* knockdown H322 cells. Scale bar, 100 µm. **e** Sphere formation efficiency of *MTHFD2* knockdown H322 cells. Experiments were performed three times and the representative results were presented. The data are represented as mean ± SD, *N* = 4. **f** mRNA expression of *SOX2* measured by quantitative RT-PCR and western blotting for SOX2 in *MTHFD2* knockdown H322 cells. The data are represented as mean ± SD, *N* = 3. **g** Tumors at the end point of the limiting dilution assay (*N* = 6). Scale bar, 1 cm. **h** mRNA expression of *MTHFD2* in MTHFD2 knockdown Adeno14-3 cells measured by quantitative RT-PCR. The data are represented as mean ± SD, *N* = 3. **i** Bright field images of tumor spheroids of *MTHFD2* knockdown Adeno 14-3 cells. Scale bar, 50 µm. **j** Sphere formation efficiency of *MTHFD2* knockdown Adeno 14-3 cells. The data are represented as mean ± SD, *N* = 3. Statistical significance is calculated with a two-tailed unpaired *t*-test; *p < 0.05, **p < 0.01

Small hairpin RNA (shRNA)-mediated knockdown of *MTHFD2* resulted in significant inhibition of cell growth in both cell lines (Figs. 2a, b and Supplementary Figures 3A and B). This growth inhibition was caused, at least, by cell cycle arrest at the G0/G1 phase (Supplementary Figure 3C). We inoculated these cells (10^7^ cells) into the flanks of severe combined immunodeficient mice. *MTHFD2* knockdown significantly inhibited tumorigenesis (Fig. 2c and Supplementary Figure 3D). Immunohistochemistry and western blot analysis confirmed the reduced expression of MTHFD2 in tumor tissues (Supplementary Figures 3E), indicating the critical role of MTHFD2 in cell growth and tumorigenesis in lung cancer. We analyzed immunohistochemistry by Ki-67 staining, a well-known cell proliferation marker, for xenograft tumors derived from *MTHFD2* knockdown and control cells (Supplementary Figure 3F). We found that *MTHFD2* knockdown tumors showed significantly lower numbers of Ki-67-positive cells, compared to control tumors. These results suggest that *MTHFD2* knockdown decreased tumor growth, in part, due to decreased cell proliferation.

In addition, to examine the toxicity of *MTHFD2* knockdown in normal cells, we knocked down *MTHFD2* in SAECs (Supplementary Figure 3G). The cell growth was modestly inhibited, though the inhibitory effects were lower ( < 45% inhibition) than those of lung cancer cell lines ( > 50% inhibition) (compare Fig. 2b, Supplementary Figure 3B vs. Supplementary Figure 3H). Thus, cancer cells appear to be more dependent on MTHFD2 for growth than normal cells.

### MTHFD2 plays critical roles in conferring cancer stem-like properties to lung cancer cells

We next examined whether MTHFD2 confers cancer stem-like properties. Tumor sphere formation is a typical property of CSCs in vitro [11, 38, 39]. We found that both cell lines formed tumor spheres (Fig. 2d and Supplementary Figure 4A) and that shRNA-mediated knockdown of *MTHFD2* significantly inhibited tumor sphere formation (Figs. 2d, e and Supplementary Figures 4A and B). Moreover, *MTHFD2* knockdown resulted in significant inhibition of the expression of SOX2, a stemness marker (Fig. 2f and Supplementary Figure 4C). We also performed an in vitro limiting dilution assay and confirmed that knockdown of *MTHFD2* significantly inhibited tumor sphere formation (Table 1). In addition, we evaluated the tumor-initiating activity, a typical feature of CSCs in vivo, by inoculating limiting dilutions of cancer cells into the flanks of immune-compromised interleukin-2-receptor γc-deficient (NSG) mice [39]. Knockdown of *MTHFD2* greatly inhibited tumorigenesis, as well as the tumor-initiating activity (Fig. 2g and Table 2).

**Table 1:** Results of limiting dilution assay of *MTHFD2* knocked down H322 cells in vitro

	Cells (per well)				Tumor-initiating cell frequency estimate	Probability
	13	25	50	100		
NCT	0/10	3/10	3/10	4/10	1/154	
*shMTHFD2* #53	0/10	0/10	0/10	2/10	1/887	0.0082**

*NCT* negative controls

**p < 0.01

**Table 2:** Results of limiting dilution assay of *MTHFD2* knocked down H322 cells in vivo

	Cells (per well)				Tumor-initiating cell frequency estimate	Probability
	10^2^	10^3^	10^4^	10^5^		
NCT	0/6	0/6	2/6	6/6	1/2.4 × 10^4^	
*shMTHFD2* #53	0/6	0/6	0/6	2/6	1/2.8 × 10^5^	0.00137**

*NCT* negative controls

**p < 0.01

In order to further clarify the effects on CSCs and differentiated cancer cells, we used the activity of aldehyde dehydrogenase (ALDH), a functional marker for CSCs [40], to sort H322 cells. We found that the ALDH^high^ cell population showed significantly higher sphere-forming activity than the ALDH^low^ cell population, confirming that the ALDH^high^ cell population is enriched with CSCs and that the ALDH^low^ cell population is mainly comprised of differentiated cancer cells (Supplementary Figure 4D). *MTHFD2* or *EGFR* was expressed in both cell populations at similar levels (Supplementary Figure 4E). This suggests that MTHFD2 and EGFR signaling play roles in both cell populations. When *MTHFD2* was knocked down, sphere-forming activity decreased in the ALDH^high^ cell population (Supplementary Figure 4F and G). When *MTHFD2* was knocked down in the ALDH^low^ cell population, cell growth decreased (Supplementary Figure 4H). Furthermore, we examined patient-derived lung adenocarcinoma cells (Adeno 14-3). When *MTHFD2* was knocked down in these cells, the sphere-forming activity was greatly decreased (Figs. 2h-j). These results indicate that MTHFD2 plays critical roles in conferring stem-like properties to lung cancer cells.

Previously, it was reported that the enzymatic activity of MTHFD2 is dispensable for cellular growth [37]. To examine whether this enzymatic activity plays roles in developing stem-like properties, we constructed a MTHFD2 mutant that lacks enzymatic activity due to a R204A mutation in the NAD-binding site as previously reported [37]. We overexpressed the mutant MTHFD2 in H322 cells using a lentiviral expression vector (Supplementary Figure 4I). To examine the stem-like properties, we analyzed sphere formation. As shown in Supplementary Figure 4J, we found that expression of the R204A MTHFD2 mutant significantly decreased sphere formation compared with control vector-transduced cells. These results suggest that the enzymatic activity of MTHFD2 is important for conferring stem-like properties in lung cancer cells.

### MTHFD2 functions are important for purine synthesis in lung cancer cells

**Fig. 3:**
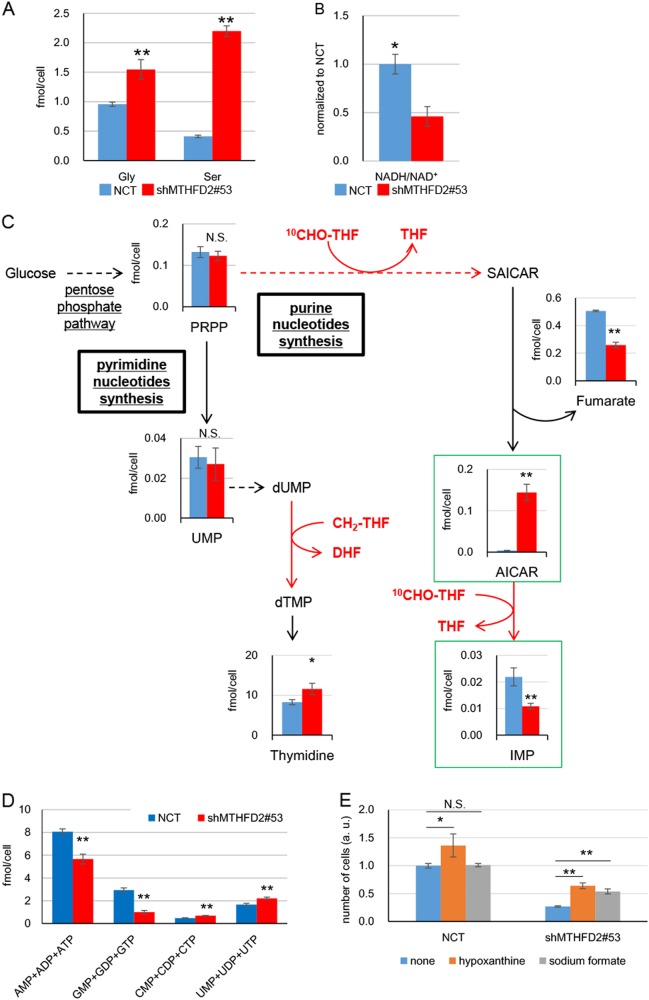
Reduction in folate-mediated 1C metabolism and purine synthesis following *MTHFD2* knockdown. **a** Intercellular concentrations of glycine (Gly) and serine (Ser). NCT negative control; shMTHFD2#53, *MTHFD2* knockdown. The data are represented as mean ± SD, *N* = 3. **b** Ratio of the amount of NADH to NAD^+^ (NADH/NAD^+^) normalized to the value in control cells. The data are represented as mean ± SD, *N* = 3. **c** Concentrations of metabolites involved in nucleotide synthesis pathway. Deep blue and red bars depict concentrations of metabolites in negative control and shMTHFD2#53 cells, respectively. Dashed lines indicate multiple reactions. Reactions transferring 1C are depicted by red lines. The data are represented as mean ± SD, *N* = 3. **d** Concentrations of purine and pyrimidine nucleotides with each base A, G, C, and U. The data are represented as mean ± SD, *N* = 3. **e** Cell proliferation assay in the presence of hypoxanthine or sodium formate. Experiments were performed three times and the representative results were presented. The data are represented as mean ± SD, *N* = 3. Statistical significance is calculated with a two-tailed unpaired *t*-test; N.S. not significant, *p < 0.05, **p < 0.01

We examined the molecular mechanisms conferring cancerous properties to MTHFD2 by performing a comprehensive analysis of the metabolome and transcriptome of H322 lung cancer cells upon *MTHFD2* knockdown. We first examined the effects on folate-mediated 1C metabolism. Although the expression level of *MTHFD2* was greatly reduced up to ~1/10 by knockdown, only a small difference was observed (< 1.5-fold) in the expression levels of all other major enzymes involved in 1C metabolism (Supplementary Figures 5A and B). In contrast, we found a > 5-fold increase in serine accumulation in *MTHFD2* knockdown cells (Fig. 3a); the amount of glycine was modestly higher (approximately 1.5-fold) upon *MTHFD2* knockdown (Fig. 3a). In addition, the ratio of NADH/NAD^+^ was significantly reduced in *MTHFD2* knockdown cells (Fig. 3b). These observations suggest that inhibition of MTHFD2-catalyzed reactions leads to reduction in the products (NADH and ^10^CHO–THF) and accumulation of substrates (NAD^+^ and CH_2_–THF; Fig. 1a in mitochondria), thereby resulting in subsequent inhibition of serine hydroxymethyltransferase 2 (SHMT2)- or glycine decarboxylase (GLDC)-mediated production of CH_2_–THF and the accumulation of serine and glycine.

We next determined the effects on the purine and pyrimidine synthesis pathways, in which 1C metabolism products are used as building blocks. Phosphoribosyl diphosphate (PRPP), a product of the pentose phosphate pathway, is used for the synthesis of purine and pyrimidine nucleotides (Fig. 3c). Two carbons are incorporated twice during the conversion of ^10^CHO–THF to THF in purine synthesis, whereas 1 C is incorporated during the conversion of CH_2_–THF to dihydrofolate in pyrimidine synthesis. We found that knockdown of *MTHFD2* did not result in any significant change in the expression levels of major enzymes involved in the conversion of PRPP to purine (Supplementary Figure 5C). In contrast, the amount of each of the following purine nucleotides was greatly reduced in *MTHFD2* knockdown cells: inosine monophosphate (IMP), the first synthesized purine nucleotide located downstream in this pathway (Fig. 3c) and IMP-derived purine nucleotides, AMP + ADP + ATP and GMP + GDP + GTP (Fig. 3d). Among PRPP and IMP, we found that AICAR was markedly accumulated in *MTHFD2* knockdown cells (Fig. 3c). AICAR is a substrate for the second reaction in the incorporation of 1C from ^10^CHO–THF, leading to the production of IMP (Fig. 3c). ^10^CHO–THF deficiency likely leads to inhibition of the reaction that produces IMP, resulting in accumulation of the substrate AICAR. We also found that the amount of fumarate was reduced in *MTHFD2* knockdown cells (Fig. 3c).

No significant change was observed in the expression levels of major enzymes involved in the reaction from PRPP to thymidine (Supplementary Figure 5C). The amount of pyrimidine nucleotides was slightly increased in *MTHFD2* knockdown cells (uridine monophosphate (UMP)-derived pyrimidine nucleotides: thymidine, CMP + CDP + CTP, and UMP + UDP + UTP; Figs. 3c, d).

We investigated whether the deficiency in purine nucleotides is responsible for the growth defects in *MTHFD2* knockdown cells. We exogenously supplemented the culture medium with the purine derivative hypoxanthine, which can be utilized for synthesis of IMP. Addition of hypoxanthine partially restored cell growth in *MTHFD2* knockdown cells (Fig. 3e). Furthermore, we examined the effects of ^10^CHO–THF and formate deficiency on the growth of *MTHFD2* knockdown cells (Fig. 1a) by adding sodium formate to the culture medium. Addition of sodium formate partially restored cell growth in *MTHFD2* knockdown cells (Fig. 3e). However, addition of the pyrimidine nucleotide thymidine did not exert any significant effect on the growth of either *MTHFD2* knockdown or control cells (Supplementary Figure 5D). These results suggest that MTHFD2 is responsible for cell growth through purine nucleotide synthesis mediated by the production of ^10^CHO–THF and formate in the mitochondria of cancer cells.

We performed metabolome analysis using another lung cancer cell line, A549 cells, to examine whether the observed effects in H322 cells were in common with A549 cells. We found marked accumulation of serine and AICAR in *MTHFD2* knockdown A549 cells, similarly observed in *MTHFD2* knockdown H322 cells (Supplementary Figure 5E).

Glutaminolysis is one of the major metabolism pathways in cancer cells [41]. Consistent with the previous report using *MTHFD2* knockdown breast cancer cells [42], glutamate (Gln), glutamic acid (Glu), and alanine, which are metabolites in glutaminolysis, were greatly accumulated in both *MTHFD2* knockdown H322 and A549 cells (Supplementary Figure 6). Hence, *MTHFD2* knockdown appears to affect glutaminolysis. Glycolysis (Supplementary Figure 7) is upstream of the nucleotide synthesis pathways (Fig. 3c). Among glycolysis metabolites, fructose-1,6-biphosphate, dihydroxyacetone-phosphate, and glyceraldehyde-3-phosphate were significantly accumulated in both *MTHFD2* knockdown H322 and A549 cells. As these metabolites are also used in the pentose phosphate pathway, inhibition of purine nucleotide synthesis may lead to accumulation of these metabolites in the pentose phosphate pathway and glycolysis in *MTHFD2* knockdown cells.

### Association between higher expression of *MTHFD2* and worse prognosis in lung adenocarcinoma patients

**Fig. 4:**
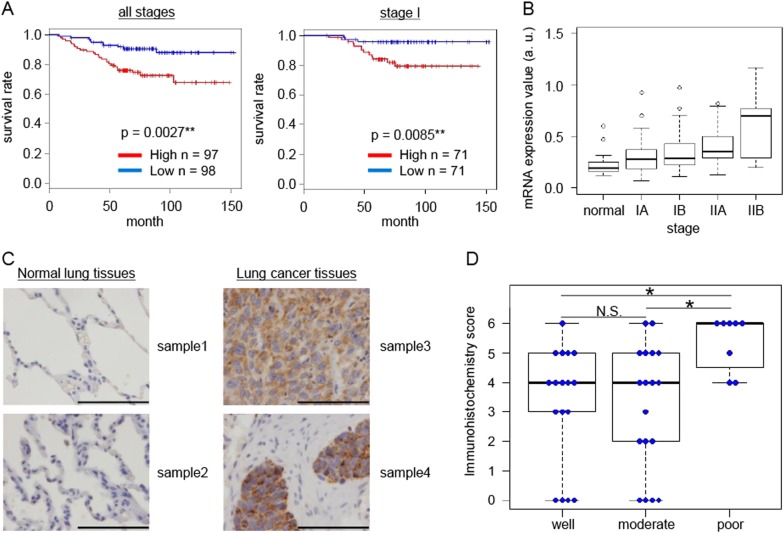
Association of higher expression of MTHFD2 with worse prognosis and malignant status of lung cancer. **a** Kaplan–Meier survival curves of patients in the NCC cohort. Red and blue lines depict survival curves of patients with cancer tissues with high and low levels of *MTHFD2* mRNA, respectively. The cutoff value was determined as the median value of the expression levels of *MTHFD2* mRNA. The *p*-values were calculated with a log-rank test. **b** Box-whisker plot of *MTHFD2* mRNA for patients in different stages (edges of boxes represent the 25th and 75th percentiles and bold lines in boxes represent the median value). Whiskers were elongated to the largest and smallest values, which are not outliers. **c** Immunohistochemistry for MTHFD2 in normal and lung cancer tissues. Scale bar, 100 µm. **d** Comparison of MTHFD2 expression levels among lung cancer tissues that show different differentiation grades on the tissue microarray. Blue circles indicate scores of each patient. Statistical significance is calculated with Steel–Dwass method. N.S. not significant; *p < 0.05; **p < 0.01

We studied the association between *MTHFD2* expression levels and the prognosis of lung adenocarcinoma patients. Analysis of the NCC cohort [35] revealed a significant association between higher expression of *MTHFD2* and worse prognosis among patients at all stages and even those with stage I (Fig. 4a). *MTHFD2* expression increased in advanced stages (Fig. 4b), and similar results were observed in the analysis of Memorial Sloan-Kettering Cancer Center (MSKCC) cohort [43] (Supplementary Figure 8). Multivariate analysis revealed that *MTHFD2* expression is an independent prognostic marker in both cohorts, with age and stage as clinical variables (Supplementary Table 1).

Examination of tissue microarrays from lung cancer patients revealed enhanced MTHFD2 expression in cancer cells compared to very low expression in normal lung tissues (Fig. 4c). Further, the expression level of MTHFD2 increased with poor differentiation grades (Fig. 4d).

### Cancer cells with acquired gefitinib resistance are dependent on MTHFD2 for cell growth

**Fig. 5:**
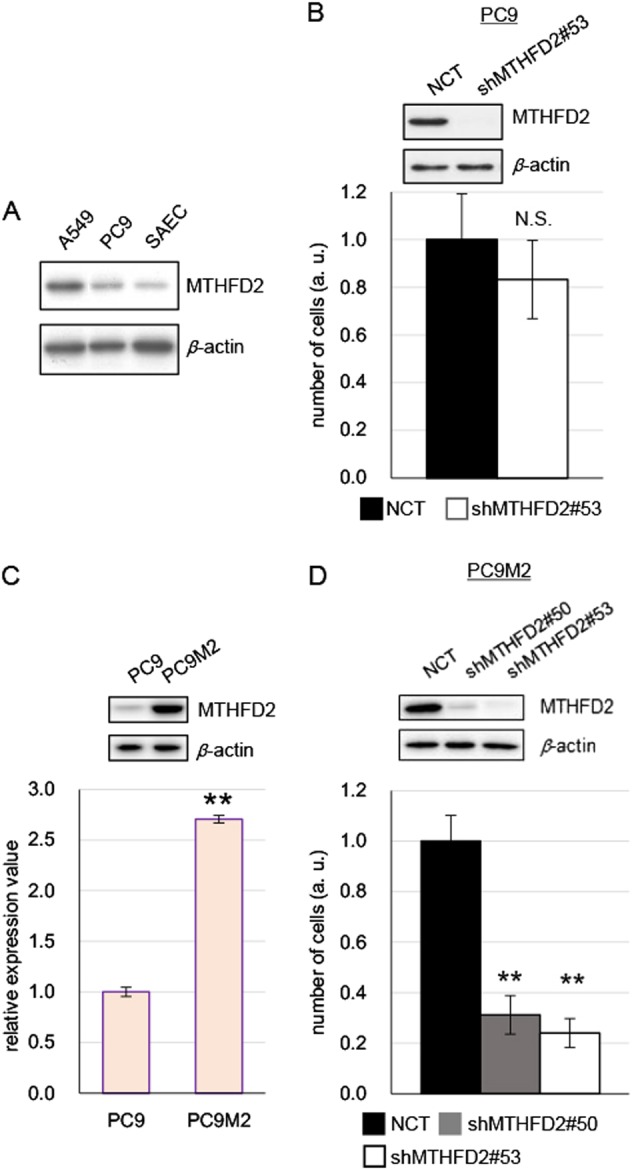
MTHFD2-dependent growth of drug-resistant PC9M2 cells. **a** Western blot for MTHFD2 in gefitinib-sensitive PC9 cells compared with A549 cells and SAECs. **b** Western blot for MTHFD2 in *MTHFD2* knockdown PC9 cells (upper panel). *β*-Actin was used as loading control. Cell proliferation assay with *MTHFD2* knockdown PC9 cells (lower panel). NCT negative control; shMTHFD2#53, *MTHFD2* knockdown (lower panel). Experiments were performed three times and the representative results were presented. The data are represented as mean ± SD, *N* = 3. **c** MTHFD2 protein (upper panel) and *MTHFD2* mRNA (lower panel) expression levels in PC9 and gefitinib-resistant PC9M2 cells. The data are represented as mean ± SD, *N* = 3. **d** Western blot for MTHFD2 in *MTHFD2* knockdown PC9M2 cells (upper panel). *β*-Actin was used as loading control. Cell proliferation assay with *MTHFD2* knockdown PC9M2 cells (lower panel). Experiments were performed three times and the representative results were presented. The data are represented as mean ± SD, *N* = 3. Statistical significance is calculated with a two-tailed unpaired *t*-test; N.S. not significant; **p < 0.01

We next evaluated whether MTHFD2 was required for the growth of drug-resistant cancer cells. The PC9 cell line is derived from the lung adenocarcinoma cells of a gefitinib-sensitive patient harboring a five-amino-acid deletion in the EGFR tyrosine kinase domain in cancer tissues [31]. We established gefitinib-resistant PC9M2 cells by culturing PC9 cells in the presence of low doses of gefitinib for several months. PC9M2 cells display properties of cancer cells with acquired drug resistance. We previously reported that there were neither additional mutations in *EGFR* nor *MET* amplification in PC9M2 cells [31]. The MTHFD2 expression level was low in PC9 cells, but was marginally higher than that in SAECs (Fig. 5a). Knockdown of *MTHFD2* did not exert significant growth inhibition in PC9 cells, consistent with the phenotype associated with low-level MTHFD2 expression (Fig. 5b). In comparison with the parental PC9 cells, PC9M2 cells showed markedly increased expression of MTHFD2 protein and mRNA levels (Fig. 5c). *MTHFD2* knockdown strongly inhibited the cell growth of PC9M2 cells (Fig. 5d). These results indicate that acquired resistance to gefitinib in PC9M2 cells is accompanied by an increase in the expression of MTHFD2 and acquired dependence on MTHFD2 for cell growth.

It has been reported that expression of *MTHFD2* is upregulated by the c-Myc transcription factor [44, 45]. We found that expression levels of *c-Myc* were upregulated in PC9M2 cells compared with those of PC9 cells (Supplementary Figure 9). It is possible that elevated c-Myc contributes to increased expression levels of MTHFD2 in PC9M2 cells.

Database analysis revealed that cancer cells carrying the resistance mutation in *EGFR* or *MET* amplification appear to depend on MTHFD2 for growth (Supplementary Table 2). Thus, cancer cells with acquired resistance to molecular targeted therapies may depend on MTHFD2 for growth in general.

### MTHFD2-mediated increase in stem-like properties and gefitinib resistance in cancer cells

**Fig. 6:**
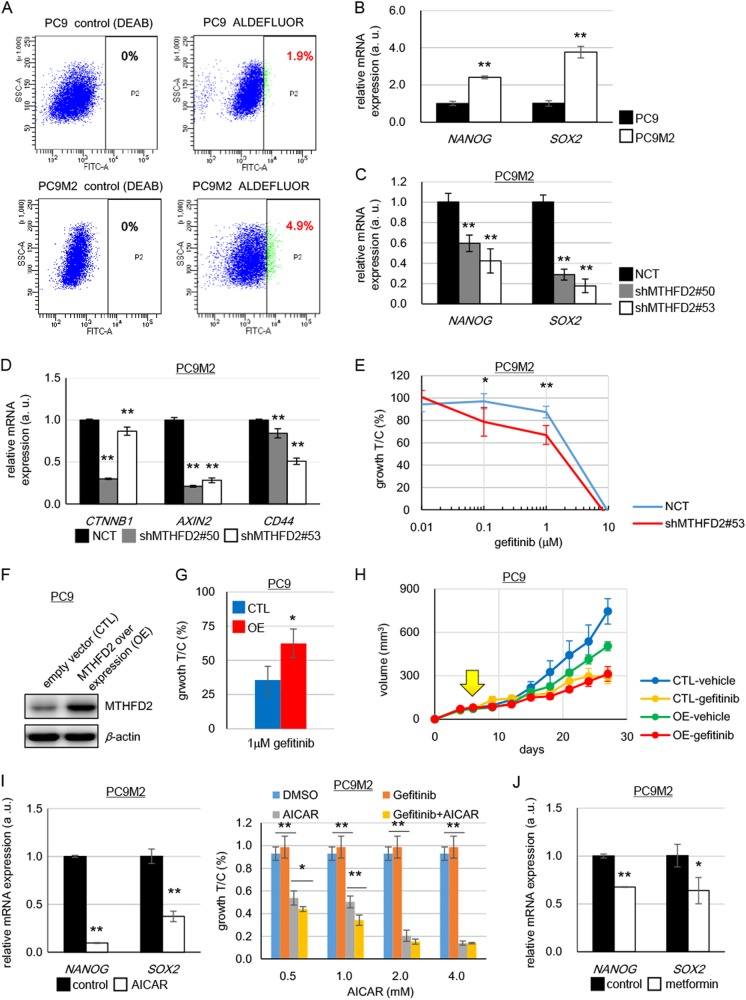
Stem-like properties and drug resistance are dependent on MTHFD2. **a** ALDH activity in PC9 and PC9M2 cells was measured using the ALDEFLUOR^®^ assay. Cells positive for ALDH are depicted with green dots as strong intensities of fluorescence measured by fluorescein isothiocyanate isomer-I (FITC) and their proportion to total cells are described in red (right panels). DEAB, an inhibitor of ALDH activity, was used for quenching and DEAB-treated cells were used as negative controls (left panels). Horizontal and vertical axes indicate intensities of ALDH activity (FITC) and complexity of cells (side scatter), respectively. **b** mRNA expression levels of stem cell markers in PC9 and PC9M2 cells were measured by quantitative RT-PCR and data are normalized by the value of PC9 cells. The data are represented as mean ± SD, *N* = 3. **c** mRNA expression levels of stem cell markers in PC9M2 cells were measured by quantitative RT-PCR and data are normalized by the value of the negative control (NCT). shMTHFD2#50, shMTHFD2#53, *MTHFD2* knockdown. The data are represented as mean ± SD, *N* = 3. **d** mRNA expression levels of *β-catenin* and its downstream targets *AXIN2* and *CD44* were measured by quantitative RT-PCR and data are normalized with the value of NCT. The data are represented as mean ± SD, *N* = 3. **e** Gefitinib sensitivity was measured by cell growth with increased doses of gefitinib in PC9M2 cells. Experiments were performed three times and the representative results were presented. The data are represented as mean ± SD, *N* = 4. **f** Western blot for MTHFD2 in empty vector-transduced control PC9 cells (CTL) and MTHFD2-overexpressing PC9 cells (OE). **g** Cell growth activity was measured with or without gefitinib treatment (1 μM). Experiments were performed three times and the representative results were presented. The data are represented as mean ± SD, *N* = 3. **h** Growth curves of tumors of CTL or OE PC9 cells. Mice were administered orally with vehicle or gefitinib (10 mg/kg/day) every day from 6 days after the inoculation (yellow arrow). Horizontal axis and vertical axis depict days after inoculation of cells and volume of tumors, respectively. The data are represented as mean ± SEM, *N* = 4. **i** mRNA expression levels of stem cell markers in PC9M2 cells were measured by quantitative RT-PCR with or without AICAR treatment (1 mM) and data are normalized by the value of control (without AICAR treatment) (left panel). Cell growth activity was measured with increased doses of AICAR treatment with or without gefitinib (1 μM) (right panel). Experiments were performed three times and the representative results were presented. The data are represented as mean ± SD, *N* = 4. **j** mRNA expression levels of stem cell markers in PC9M2 cells were measured by quantitative RT-PCR with or without metformin treatment (4 mM) and data are normalized by the value of control (without AICAR treatment). The data are represented as mean ± SD, *N* = 3. Statistical significance is calculated with a two-tailed unpaired *t*-test; N.S. not significant, *p < 0.05, **p < 0.01

We investigated the stem-like properties in PC9M2 cells with acquired drug resistance and the parental drug-sensitive PC9 cells. Because PC9 and PC9M2 cells did not show clear sphere formation, we chose to analyze the activity of ALDH, the functional marker for CSCs [40]. We found that PC9M2 cells showed a significant increase in ALDH activity (4.9%) compared with PC9 cells (1.9%) (Fig. 6a). In addition, PC9M2 cells exhibited an increase in the expression of the stemness-related transcription factors *NANOG* and *SOX2* compared with PC9 cells (Fig. 6b). Knockdown of *MTHFD2* in PC9M2 cells using shRNA resulted in a decrease in the expression of *NANOG* and *SOX2* (Fig. 6c).

Elevated expression of the components of the β-catenin pathway is responsible for gefitinib resistance in PC9M2 cells [31]. *MTHFD2* knockdown in PC9M2 cells resulted in a significant decrease in the expression of *β-catenin*, *Axin2*, and *CD44*, downstream targets in the β-catenin pathway (Fig. 6d). Consistent with this result, we found that sensitivity to gefitinib was restored in *MTHFD2* knockdown PC9M2 cells (Fig. 6e). These results indicate that enhanced expression of MTHFD2 plays important roles in inducing stem-like properties and drug resistance in PC9M2 cells.

We next examined whether MTHFD2 overexpression alone confers resistance to gefitinib. We stably overexpressed MTHFD2 in PC9 cells using a lentiviral expression vector (Fig. 6f) and found that PC9 cells overexpressing MTHFD2 showed resistance to gefitinib in vitro (Fig. 6g). Furthermore, we performed in vivo xenograft experiments. We inoculated PC9 cells overexpressing MTHFD2 and control vector-transduced PC9 cells in the flanks of NSG mice and observed tumorigenesis with or without oral administration of gefitinib. The control PC9 cells induced rapidly growing tumors, and administration of gefitinib greatly inhibited tumor growth. In contrast, PC9 cells overexpressing MTHFD2 induced relatively slow growing tumors; however, the tumors showed more resistance to gefitinib, as compared to control PC9 cells (two-way analysis of variance, *p* < 0.05) (Fig. 6h and Supplementary Figures 10A and B). Slow growing tumors with gefitinib resistance are similarly observed in PC9M2 cells [31]. Thus, overexpression of MTHFD2 conferred resistance to gefitinib.

To explore the molecular mechanisms that determine how MTHFD2 confers drug resistance and stem-like properties to cancer cells, we focused on AICAR, because it was **t**he metabolite that showed the greatest increase upon *MTHFD2* knockdown (Fig. 3c and Supplementary 5E). We found that treatment with AICAR greatly decreased the expression levels of *NANOG* and *SOX2* in PC9M2 (Fig. 6i, left panel). We also found that treatment with AICAR decreased cell growth in a dose-dependent manner as previously reported (Fig. 6i, right panel) [26, 46]. Although treatment with 1 μM of gefitinib did not show a significant change in growth activity in PC9M2 cells, combined treatment with gefitinib (1 μM) and AICAR decreased cell growth greater than AICAR alone-treated cells (Fig. 6i, right panel). This result indicates that treatment with AICAR restored gefitinib sensitivity in PC9M2 cells. The fact that AICAR is a physiological activator of AMPK suggests that AMPK activated by AICAR may reduce the stem-like properties. To test this hypothesis, we treated the cells with metformin, a pharmacological activator of AMPK [28]. We found that treatment with metformin significantly decreased the expression levels of *NANOG* and *SOX2* in PC9M2 cells; however, the effects were not as strong as those of AICAR treatment (Fig. 6i, left panel and 6j). This is consistent with the notion that the AICAR-induced decrease in cancer stem-like properties is partly mediated by the activation of AMPK. These results suggest that active 1C metabolism maintains a low amount of intracellular AICAR and low levels of AMPK activity, which play important roles in conferring drug resistance and stem-like properties to cancer cells.

## Discussion

In this study, we demonstrated that MTHFD2-mediated mitochondrial 1C metabolism plays critical roles in cancer cell growth and confers drug resistance and cancer stem-like properties, which are thought to be responsible for tumor recurrence and poor prognosis. Some cancer cells with acquired resistance to gefitinib showed increased dependence on MTHFD2, as evidenced by the increase in MTHFD2 expression. Moreover, overexpression of MTHFD2 alone in gefitinib-sensitive cancer cells conferred gefitinib resistance. These results provide evidence that targeting MTHFD2 is a promising strategy to eradicate tumors and prevent recurrence. We also elucidated the underlying mechanistic basis by which MTHFD2 promoted cell growth by producing purines. Concomitantly, MTHFD2 conferred drug resistance and cancer stem-like properties by depleting the intracellular pool of the purine intermediate AICAR, which contributed to the decrease in stemness and increase in drug sensitivity.

The role of AICAR in conferring drug resistance and cancer stem-like properties is reported for the first time in this study, to the best of our knowledge. AICAR not only acts as a purine intermediate in the synthesis of purines, but has multiple functions. Several reports show that treatment with AICAR inhibits cancer cell proliferation [26, 46]. Although studies indicate that the functions of AICAR are partly mediated by activation of AMPK, AICAR also inhibits cancer cell proliferation independent of AMPK [46]. AICAR induces differentiation of embryonic stem cells [47]. We showed that treatment with AICAR or metformin reduces cancer stem-like properties in gefitinib-resistant cells, although the effects of AICAR seemed to be stronger than those of metformin. Further research studies are needed to uncover the precise molecular mechanisms.

We found that PC9M2 cells with acquired gefitinib resistance, without additional mutations in *EGFR* or *MET* amplification, showed increased stemness. This is consistent with the notion that CSCs are resistant to a variety of stressful conditions, including cancer therapy treatment conditions. The Wnt–β-catenin pathway is crucial for the maintenance of normal stem cells and CSCs in the intestine and colon [48]. Thus, increased expression of the components of the β-catenin pathway may confer cancer stem-like properties to PC9M2 cells. We found that the expression levels of several β-catenin pathway-related genes, *β-catenin*, *Axin*, and *CD44*, were reduced in *MTHFD2* knockdown PC9M2 cells. This suggests that MTHFD2 is required for maintenance of stemness induced by the β-catenin pathway. Thus, MTHFD2-mediated mitochondrial 1C metabolism may be a general mechanism involved in the maintenance of stemness in cancer cells.

The expression levels of MTHFD2 were clearly correlated with poor prognosis, advanced stage, as well as undifferentiated grades, and the expression level served as an independent prognostic marker in lung adenocarcinoma. This finding provides additional evidence that MTHFD2 contributes to malignancy in lung cancer. In addition, stage I lung adenocarcinoma patients can be stratified into a good prognostic group with low-level *MTHFD2* expression and a poor prognostic group with high-level *MTHFD2* expression in cancer tissues. Because patients with a poor prognosis have a high risk of recurrence after surgery [33], MTHFD2-targeting therapies could be added to their treatment regimen to prevent recurrence when the expression level of MTHFD2 is high in the resected cancer tissues.

We found that PC9 cells with low levels of MTHDF2 expression did not show a significant change in cell growth by *MTHFD2* knockdown, but *MTHFD2* knockdown greatly inhibited growth in PC9M2 cells in which MTHFD2 is highly expressed. These results suggest that cancer cells with acquired gefitinib resistance develop dependence on MTHFD2 for cell growth. When cells are dependent on MTHFD2, this enzyme may become rate limiting for the synthesis of sufficient amount of purine nucleotides, promoting rapid growth of cancer cells and CSCs. When MTHFD2 expression is low, other enzymes such as cytosolic MTHFD1 may compensate for insufficient levels of MTHFD2 (Fig. 1a). In a recent study, *MTHFD2* knockout cell lines were generated using the clustered regularly interspaced short palindromic repeats (CRISPR)-Cas9 system [49]. The authors reported that these cells use a compensatory cytosolic pathway and not the mitochondrial pathway, suggesting that cells adapted to the low expression levels of MTHFD2 are able to survive using alternate pathways.

Activation of the EGFR tyrosine kinase induces MTHFD2 expression in some cancer, as well as normal epithelial cells. A recent study reported that the oncogenic transcription factor Myc is involved in the expression of MTHFD2 in leukemia cells and tumor-initiating cells in glioblastoma [50]. EGF stimulation strongly induces Myc [51], and therefore, EGF-induced expression of MTHFD2 may be mediated by Myc. A very recent study showed that Myc contributes to the expression of metabolic enzymes from PRPP to AMP and GMP in purine synthesis for the maintenance of brain tumor-initiating cells [52]. Both folate-mediated 1C metabolism and the purine synthesis pathway, beginning with the formation of PRPP, seem to be important for the maintenance of CSCs or tumor-initiating cells.

Collectively, it is possible that MTHFD2-mediated 1C metabolism plays important roles for conferring drug resistance and cancer stem-like properties not only in lung cancer but also in other types of malignancy including solid tumors and leukemia.

## Materials and methods

### Cell lines

Lung cancer cell lines used in these experiments were purchased from the American Type Culture Collection (ATCC) and kind gifts from Jun Yokota and Takashi Kohno. Adeno 14-3 cells were established from adenocarcinoma tissues of a lung cancer patient. Further, they were cultured in RPMI-1640 (Nacalai Tesque) with 10% (vol/vol) fetal bovine serum (FBS) (ThermoFisher Scientific), 100 units/mL penicillin, and 100 μg/mL streptomycin (Nacalai Tesque). SAECs were purchased from Lonza and cultured in SAGM BulletKit (Lonza). HEK293T cells (Lenti-X 293T Cell Line) were purchased from Clontech and cultured in Dulbecco’s modified Eagle’s medium (DMEM; Nacalai Tesque) supplemented with 10% (vol/vol) FBS (ThermoFisher Scientific) and 100 units/mL penicillin, and 100 μg/mL streptomycin (Nacalai Tesque). All cells were cultured at 37 °C in 5% CO_2_.

### Lentiviral vectors for constructing lentivirus containing a vector to produce shRNA for MTHFD2

For the shRNA-producing vector, we used the pLKO.1 vector (Sigma Aldrich). As a negative control vector, we used the non-mammalian shRNA control plasmid SHC002 (Sigma Aldrich). The sequences of the shRNA for MTHFD2 are as follows:

shMTHFD2#50: 5′-CCGGCGAATGTGTTTGGATCAGTATCTCGAGATACTGATCCAAACACATTCGTTTTTG-3′; shMTHFD2#53: 5′-CCGGGCAGTTGAAGAAACATACAATCTCGAGATTGTATGTTTCTTCAACTGCTTTTTG-3′.As packaging plasmids, we used pCMV-VSV-G-RSV-Rev and pCAG-HIVgp, which were kind gifts from H. Miyoshi (RIKEN, Tsukuba, Japan).

### Lentiviral vectors for constructing lentivirus containing a vector to produce MTHFD2

Full-length complementary DNA (cDNA) of MTHFD2 (NM_006636.3) was amplified from tumor cDNA by PCR and then inserted into lentivirus vector, pLenti-6/V5-DEST (Invitrogen). By using Sanger sequencing, the integrity of inserted cDNA was verified. As packaging plasmids, we used pLP1, pLP2 and pLP_VSVG (Invitrogen). To generate dehydrogenase-dead MTHFD2, PrimeSTAR Mutagenesis Basal Kit (Takara, R046A) was used to mutate Arg to Ala in the following sequence: KNVVVAGRSKN [37, 53].

### Transduction of cells with lentiviral vectors

Viral solution was produced by co-transfecting plasmids in HEK293T cells by using lipofectamine and plus reagent (ThermoFisher Scientific), as previously described [54]. Cells were cultured with viral solution and then incubated overnight at 37 °C in 5% CO_2_. For selection of cells stably expressing shRNA for MTHFD2, cells were cultured in medium containing 2.0 μg/mL puromycin with respect to A549 cells and 2.5 μg/mL puromycin for H322, PC9, and PC9M2 cells. For selection of cells stably expressing MTHFD2, cells were cultured in medium supplemented with 10 mg/mL blasticidin (Invitrogen). The mass cultures of MTHFD2-expressing cells were used for experiments.

### Cell growth assay

Cells were plated at a density of 5.0 × 10^3^–2.0 × 10^4^ cells/well (depending on the growth rate of the cell lines) in 12-well plates and cultured for 4–6 days. The medium was replaced with new medium once every 2 days except for the assay with PC9M2 cells. For assays with metabolite supplementation, Minimum Essential Medium Eagle with Earle's Salts and L-Gln (MEM) (Nacalai Tesque) with non-essential amino acids and 10% (vol/vol) dialyzed FBS (ThermoFisher Scientific) was used to exclude the effects of metabolites in FBS. Final concentrations of supplemented metabolites were 30 μM for hypoxanthine and thymidine and 1.0 mM for sodium formate.

### Gefitinib sensitivity analysis

PC9 cells or PC9M2 cells were plated onto 96-well plate. Cells were treated with gefitinib (Selleck chemicals) at the indicated concentrations. After 72-h treatment, the cell viability was measured using CellTiter 96 AQueous One Solution Cell Proliferation Assay (Promega).

### EGF stimulation

SAECs and H322 cells were stimulated with 11 ng/mL, 20 ng/mL, or 100 ng/ml EGF (Merck Millipore), respectively, after overnight starvation. The final concentration of gefitinib (Selleck Chemicals) was 1.0 μM.

### Sphere formation assay

The sphere formation assay was performed as described [12]. Briefly, cells were plated as single cells on an ultralow attachment 24-well plate (Corning) at a density of 2000 cells/well. They were grown in sphere culture medium (SCM), which consisted of DMEM/F-12 medium (ThermoFisher Scientific) supplemented with 20 ng/mL EGF (Merck Millipore), 20 ng/mL basic fibroblast growth factor (PeproTech), B27 (ThermoFisher Scientific), and 4 μg/mL heparin (Stem Cell Technologies). Spheres with a diameter > 75 μm were counted after 4 days.

### Microarray

Total RNA was extracted using Trizol reagent (ThermoFisher Scientific) and quality was assessed using an Agilent 4200 TapeStation (Agilent Technologies). All samples showed RNA Integrity Numbers of >8.9 and were subjected to microarray analysis according to the manufacturer’s instructions. In brief, RNA samples were labeled using the Low Input Quick Amp Labeling Kit (Agilent Technologies). Labeling of 200 ng total RNA was performed using cyanine 3-CTP. Hybridization was performed using the Gene Expression Hybridization Kit (Agilent Technologies). complementary ribonucleic acid (cRNA) samples (600 ng) were fragmented (30 min at 60 °C) and then hybridized to the SurePrint G3 Human GE microarray 8 × 60K Ver. 3.0 (G4851C, Agilent Technologies) in a rotary oven (65 °C for 17 h). Slides were washed in Agilent Gene Expression Wash Buffers 1 and 2 (Agilent Technologies) and scanned immediately after washing on the Agilent DNA Microarray Scanner (G2539A) using the one color scan setting for 1 × 60k array slides (Scan Area 61 × 21.6 mm, Scan resolution 3 μm, Dye channel was set to Green, and Green PMT was set to 100%). The scanned images were analyzed with Feature Extraction Software 11.0.1.1 (Agilent Technologies) using default parameters (protocol AgilentG3_GX_1Color and Grid: 072363_D_F_20150612) to obtain background-subtracted and spatially detrended processed signal intensities. To adjust for differences in the probe intensity distribution across different arrays, gene expression values were normalized with GeneSpring software (Agilent Technologies) using the 75th percentile value.

### Metabolite quantification

Metabolites were collected as follows. Cells were washed with 5% mannitol and lysed with methanol containing three internal standards (final, 25 μM each); l-methionine sulfone (Wako), 2-morpholinoethanesulfonic acid monohydrate (Dojindo), and d-camphor-10-sulfonic acid sodium salt (Wako). After vortexing, 400 μL of CHCl_3_ and 200 μL of water were added to the supernatants. After mixing, samples were centrifuged at 10,000 × *g* for 3 min at 4 °C. Then, 400 μL of the aqueous layer was applied to a HMT 5-kDa ultrafiltration tube. Samples were centrifuge filtered at 9100 × *g* for 3 h at 4 °C. Finally, 320 μl of filtrates was centrifuge concentrated for 2 h at 40 °C. Prior to detection, samples were dissolved in 25 μL of water containing two internal standards (final, 200 μM each); 3-aminopyrrolidine (Sigma Aldrich) and 1,3,5-benzenetricarboxylic acid (Wako). Capillary electrophoresis/mass spectrometry was run as described in previous studies [55–59].

### Western blotting

Cells were lysed in lysis buffer as described [11]. Primary antibodies against MTHFD2 (Abcam, ab56772), SOX2 (Cell Signaling, #3579), and *β*-actin (Merck Millipore, MAB1501) were used. Horseradish peroxidase-conjugated secondary antibodies specific to mouse or rabbit IgG were used (GE). Signals were detected with Immobilon (Merck Millipore).

### Quantitative RT-PCR

Total RNA was prepared by using TRIzol Reagent (ThermoFisher Scientific) or RNeasy Mini Kit (Qiagen) and then reverse transcribed into cDNA using the High Capacity cDNA Reverse Transcription kit (ThermoFisher Scientific). Quantitative RT-PCR of *MTHFD2* and 18S rRNA were performed with Taqman probe (Applied Biosystems) or with following primers for *MTHFD2* (forward: 5′-CAGCTGCAGTTGTGGGAAT-3′, reverse: 5′-GGAGGCCATCTACATTATCATCA-3′), *NANOG* (forward: 5′-ATGCCTCACACGGAGACTGT-3′, reverse: 5′-CAGGGCTGTCCTGAATAAGC-3′),　*SOX2* (forward: 5′-GGGGGAATGGACCTTGTATAG-3′, reverse: 5′-GCAAAGCTCCTACCGTACCA-3′), *CTNNB1* (forward: 5′-GCTTTCAGTTGAGCTGACCA-3′, reverse: 5′-CAAGTCCAAGATCAGCAGTCTC-3′), *CD44* (forward: 5′-GCAGTCAACAGTCGAAGAAGG-3′, reverse: 5′-TGTCCTCCACAGCTCCATT-3′), *AXIN2* (forward: 5′-GCTGACGGATGATTCCATGT-3′, reverse: 5′-ACTGCCCACACGATAAGGAG-3′), *EGFR* (forward: 5′-GATCCAAGCTGTCCCAATG-3′, reverse: 5′-GCACAGATGATTTTGGTCAGTT-3′), *MYC* (forward: 5′-CACCAGCAGCGACTCTGA-3′, reverse: 5′-GATCCAGACTCTGACCTTTTGC-3′) and *ACTB* (forward: 5′-AAGTCCCTTGCCATCCTAAAA-3′, reverse: 5′-ATGCTATCACCTCCCCTGTG-3′). TaqMan Fast Universal PCR Master Mix (2 × ), no AmpErase UNG (ThermoFisher Scientific) and Fast SYBR Green Master Mix (ThermoFisher Scientific) were exploited for reactions with Taqman probes and other primers, respectively. Reactions were performed in an Applied Biosystems StepOne real-time PCR system or ViiA7 RT-PCR system.

### Tumor growth assay

A total of 1.0 × 10^7^ cells expressing the indicated constructs were suspended in phosphate-buffered saline (PBS), and 100 μL of the cell mixture was injected subcutaneously into 8-week-old female severe combined immunodeficient mice (CLEA Japan, Inc.). Tumor volume was measured using the following formula: V = 4/3 × π*(S/2) × (L/2)^2^, where S and L are the lengths of the minor axis and major axis, respectively. PC9 cells were injected subcutaneously into the flanks of 6-week-old female NSG mice (5 × 10^6^ cells/mouse) (*N* = 4).

From 6 days after inoculation of tumor cells, mice were randomly assigned either control or gefitinib treated group, and oral administration of gefitinib (10 mg/kg/day) or vehicle was conducted daily. No blind experiments were done.

### Limiting dilution assay in vivo

Cells were suspended in PBS, and 100 μL of the cell mixture was injected subcutaneously into 8-week-old female NSG mice (Charles River). No blind experiments were done.

### Immunocytochemistry

Cells were fixed with 3% paraformaldehyde/5% sucrose for 15 min. Subsequently, cells were permeabilized with 50% methanol/50% acetone for 20 min at −20 °C and with 0.2% NP-40 for 10 min at room temperature. Cells were blocked with 5% normal goat serum (Wako)/5 mg/mL bovine serum albumin (BSA; Nacalai) for 15 min. As a mitochondrial marker, we used an OXCT1 antibody (Proteintech, 12175-1-AP), and a MTHFD2 antibody (abcam, ab56772) was used at 1:100 dilutions. Samples were reacted with the first antibody overnight at 4 °C. As secondary antibodies, goat anti-mouse IgG (H + L) cross-adsorbed secondary antibody, Alexa Fluor 568 (ThermoFisher Scientific, A-11004, 1:1000 dilution) and goat anti-rabbit IgG-FITC (Santa Cruz Biotechnology, sc-2012, 1:100 dilution) were used. Samples were reacted with secondary antibodies for 60 min. Finally, nuclei were stained with 0.2 μg/mL 4,6-diamidino-2-phenylindole (DAPI; Dojindo, 340-07971). Slides were mounted with fluorescence mounting medium (Agilent, S3023) and photographed by Zeiss LSM510 confocal microscopy.

### Immunohistochemistry

Tumors were fixed in formalin and embedded in paraffin. Tissue sections were deparaffinized in xylene, rehydrated in a graded series of ethanol, and subjected to antigen retrieval by heat treatment in EDTA buffer, pH 9.0. Anti-MTHFD2 (Abcam, ab56772) was used as the primary antibody. Biotinylated secondary antibody treatment was then performed, and signals were detected with diaminobenzidine in Tris-HCl and H_2_O_2_. Then, samples were stained with hematoxylin, dehydrated in a graded series of ethanol, cleared with xylene, and coverslipped. With respect to Ki-67 analysis, the sections were cut at 4 μm thickness. The deparaffinized slides were heated in 10 mmol/L citrate buffer (pH 6.0) for 20 min at 121 °C. After endogenous peroxidase activity was blocked using 0.3% H_2_O_2_ PBS for 30 min, the sections were incubated with Blocking One Histo (Nacalai Tesque) for 15 min. The sections were incubated with anti-Ki-67 antibody (Dako, m7249) using 0.1% BSA PBS overnight in a humidified box at 4 °C. The samples were examined using a microscopy system (BZ-X700). Images were obtained with the BZ-X700 microscope and quantified using the Keyence Analysis Software (Keyence). Ki-67+ cells in the area of intact tumor sites were measured in three randomly chosen visual fields of each tumor at 100× magnification.

### Public microarray data

Microarray data and clinical covariates of the NCC cohort and MSKCC cohort were downloaded from the GEO database (GSE31210 [33, 35] and the caArray database (data of Shedden et al. [43]), respectively.

### Tissue microarray

The lung cancer tissue microarray (CC5) and normal tissue microarray (AC1) were purchased from Super Bio Chips laboratories (http://www.tissue-array.com/). Tissue microarrays accommodated 49 primary lung cancer cases and 2 normal lung tissues, totally. The demographic and clinicopathological details of patients and tumors are provided in the manufacturer’s websites. Immunohistochemical evaluation of MTHFD2 were performed by a pathologist through light microscopic observations in a blinded fashion.

### Measurement of ALDH enzymatic activity

Cells were sorted by FACS Aria II (BD Biosciences). The ALDH enzymatic activity was measured using an ALDEFLUOR^®^ kit (STEMCELL TECHNOLOGIES) according to the manufacturer’s instructions [60]. Briefly, the harvested cells were incubated in ALDEFLUOR^®^ assay buffer containing the ALDH substrate, BODIPY–aminoacetaldehyde (BAAA), for 40 min at 37 °C. Once inside the cell, BAAA is converted into BODIPY–aminoacetate (BAA^−^) by the intracellular ALDH enzymatic activity. Intracellular BAA^−^ induces fluorescence in the cells. An aliquot of ALDEFLUOR^®^-stained cells was immediately quenched with 1.5 mM diethylaminobenzaldehyde (DEAB, ALDH inhibitor) and used as a negative control. Cells were analyzed using FACSCanto II flow cytometer (BD Biosciences) and data evaluated with FACSDIVA software (BD Biosciences).

### Cell culture with AICAR or metformin

One day after seeding of PC9M2 cells, AICAR (Tokyo Chemical Industry) (1 mM) or metformin hydrochloride (metformin) (Wako) (4 mM) was added to the medium. RNA was harvested at 90 h after addition of AICAR or metformin. With respect to growth assay, the cell viability was measured using CellTiter 96 AQueous One Solution Cell Proliferation Assay (Promega) at 72 h after addition of AICAR or/and gefitinib.

### Structure modeling

The structure of human MTHFD2 was modeled with Swiss-Model software [61–63] by using the crystal structure of FolD, a homolog of MTHFD2, of *E. coli* (pdb accession number: 1B0A [64]). Root mean square deviation was 0.069. The modeled structure of human MTHFD2 and its druggable binding sites were detected by PockDrug-Server.

### Statistical analysis

Values are presented as means ± SD or means ± SEM. Each statistical method is written in figure legend. Values of *p* < 0.01-0.05 (*), *p* < 0.001-0.01 (**) or *p* < 0.001(***) were considered significant. Tumor-initiating cell frequency was calculated with ELDA software (http://bioinf.wehi.edu.au/software/elda/index.html), provided by the Water and Eliza Hall Institute [65].

### Study approval

Patient-derived lung adenocarcinoma cells were obtained from Kanazawa University Hospital. Written informed consent was received from all participants before inclusion in the study. The study was approved by the Institutional Review Board of Kanazawa University.

Mice were handled according to the guidelines of the Institute of Medical Science at The University of Tokyo and Kanazawa University. The experiments were approved by the Committees for Animal Research at the Institute of Medical Science at The University of Tokyo and Kanazawa University.

## Supplementary material

Sup Fig1, Sup Fig2, Sup Fig3, Sup Fig4, Sup Fig5, Sup Fig 6, Sup Fig 7, Sup Fig 8, Sup Fig 9, Sup Fig 10, Sup Table 1, Sup Table 2

Figure legends for Supplementary figures clean

## Data Availability

Raw and processed microarray data have been deposited in the GEO repository. The accession number is GSE84007
