# Supplementary material for: Cancer stem-like properties and gefitinib resistance are dependent on purine synthetic metabolism mediated by the mitochondrial enzyme MTHFD2
Source: Oncogene. 2018 Dec 7;38(14):2464–81. doi: 10.1038/s41388-018-0589-1 (PMC6484769; doi:10.1038/s41388-018-0589-1)
Supplement: Supplementary file 1 — Sup Fig1, Sup Fig2, Sup Fig3, Sup Fig4, Sup Fig5, Sup Fig 6, Sup Fig 7, Sup Fig 8, Sup Fig 9, Sup Fig 10, Sup Table 1, Sup Table 2 [file 41388_2018_589_MOESM1_ESM.pdf]

Supplementary Figure 1

A

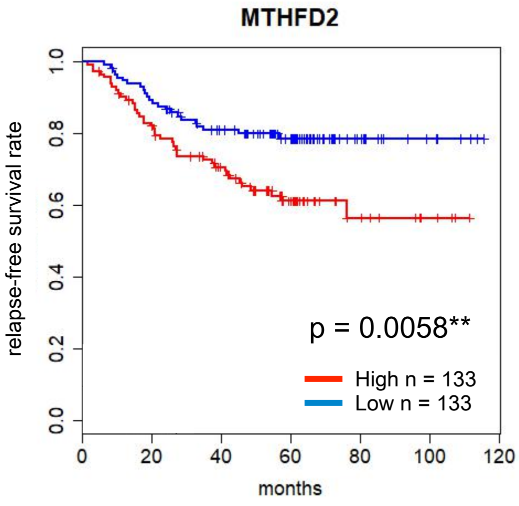

B

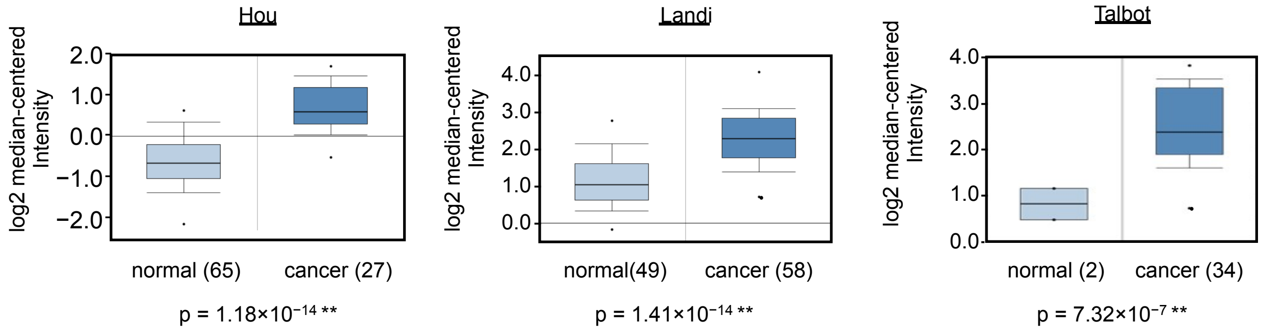

C

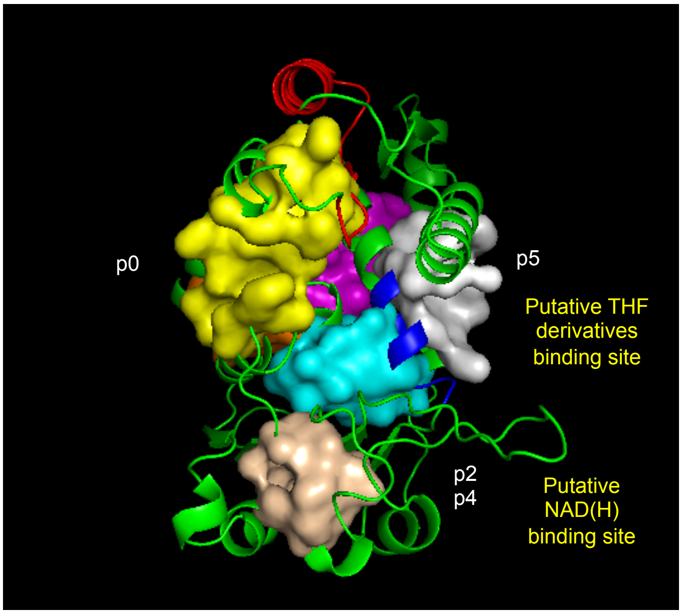

D

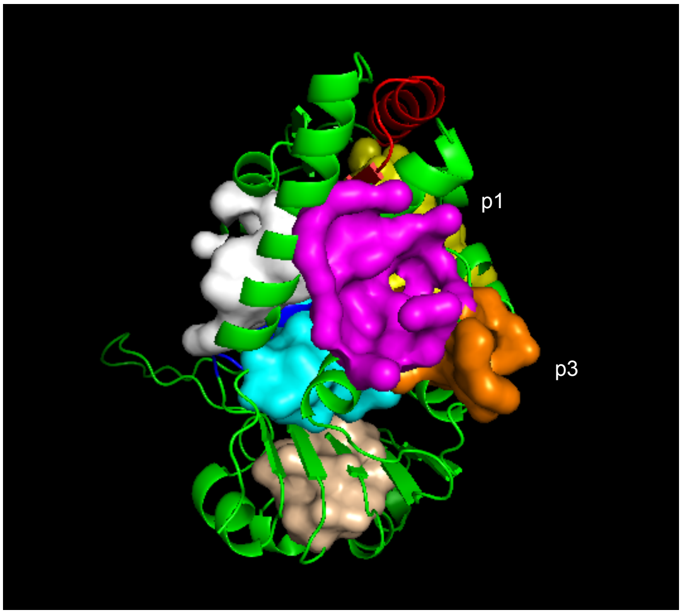

Supplementary Figure 2

A

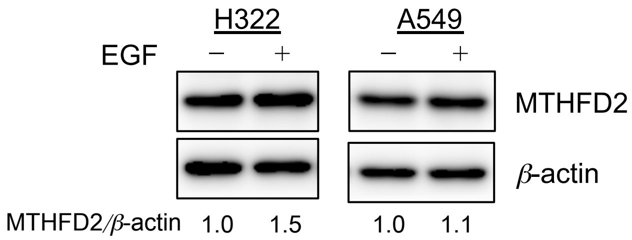

B

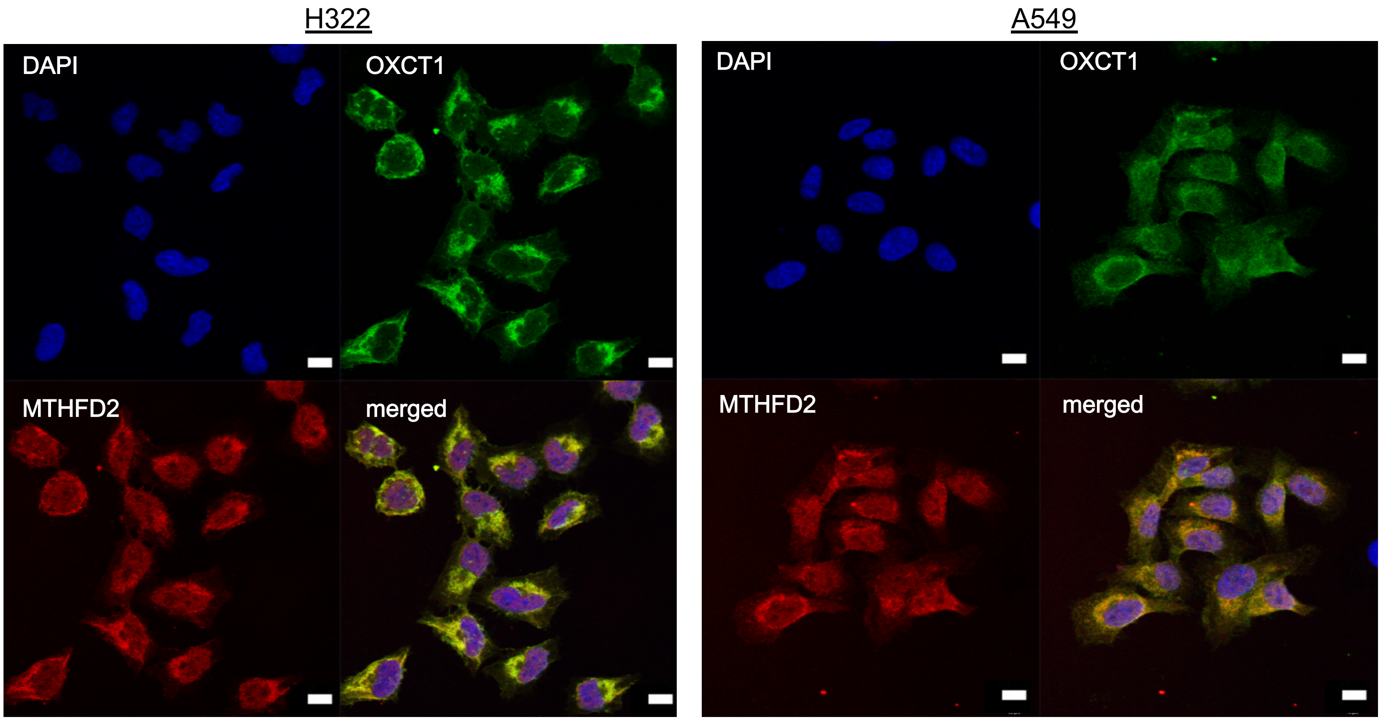

Supplementary Figure 3

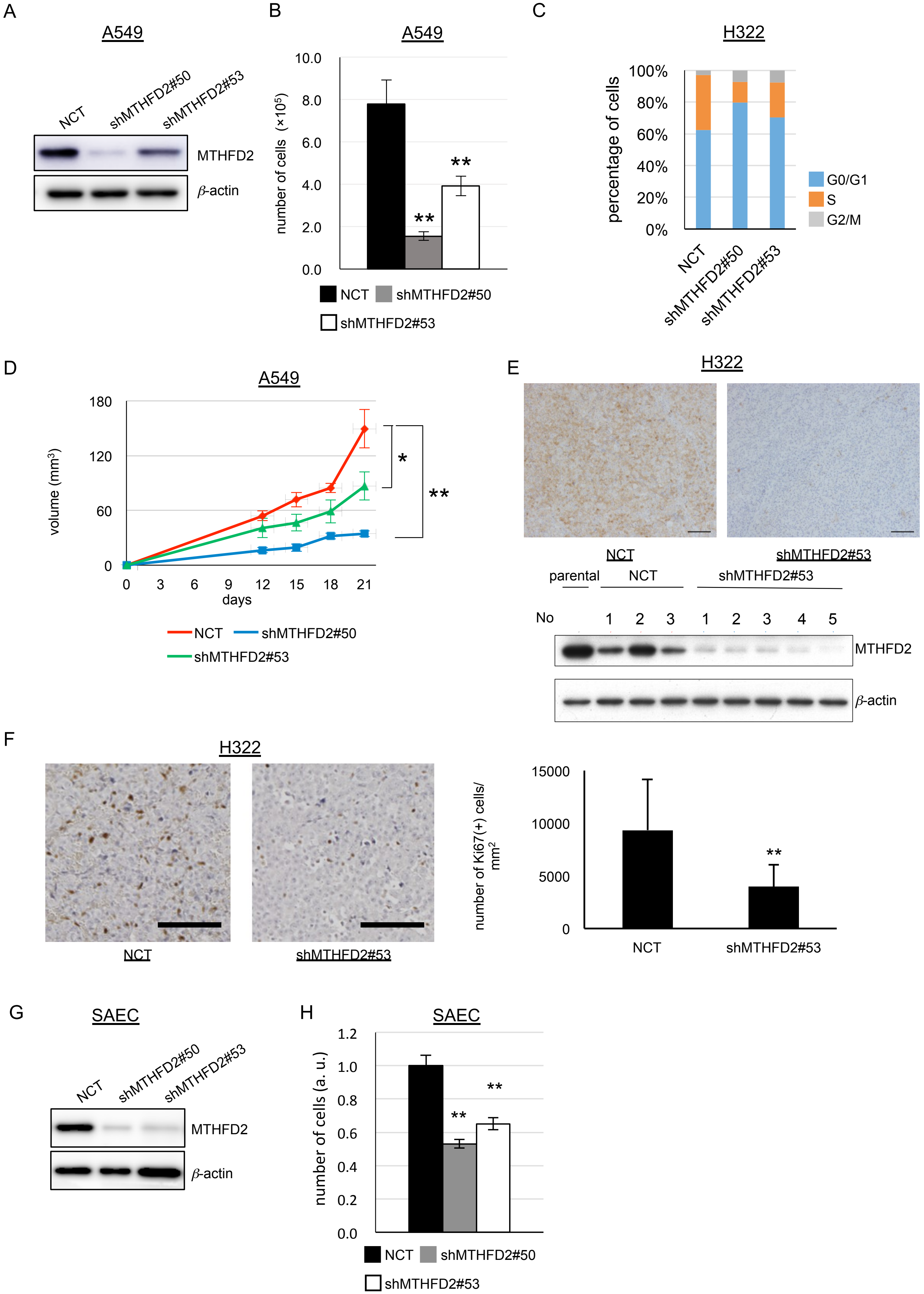

Supplementary Figure 4

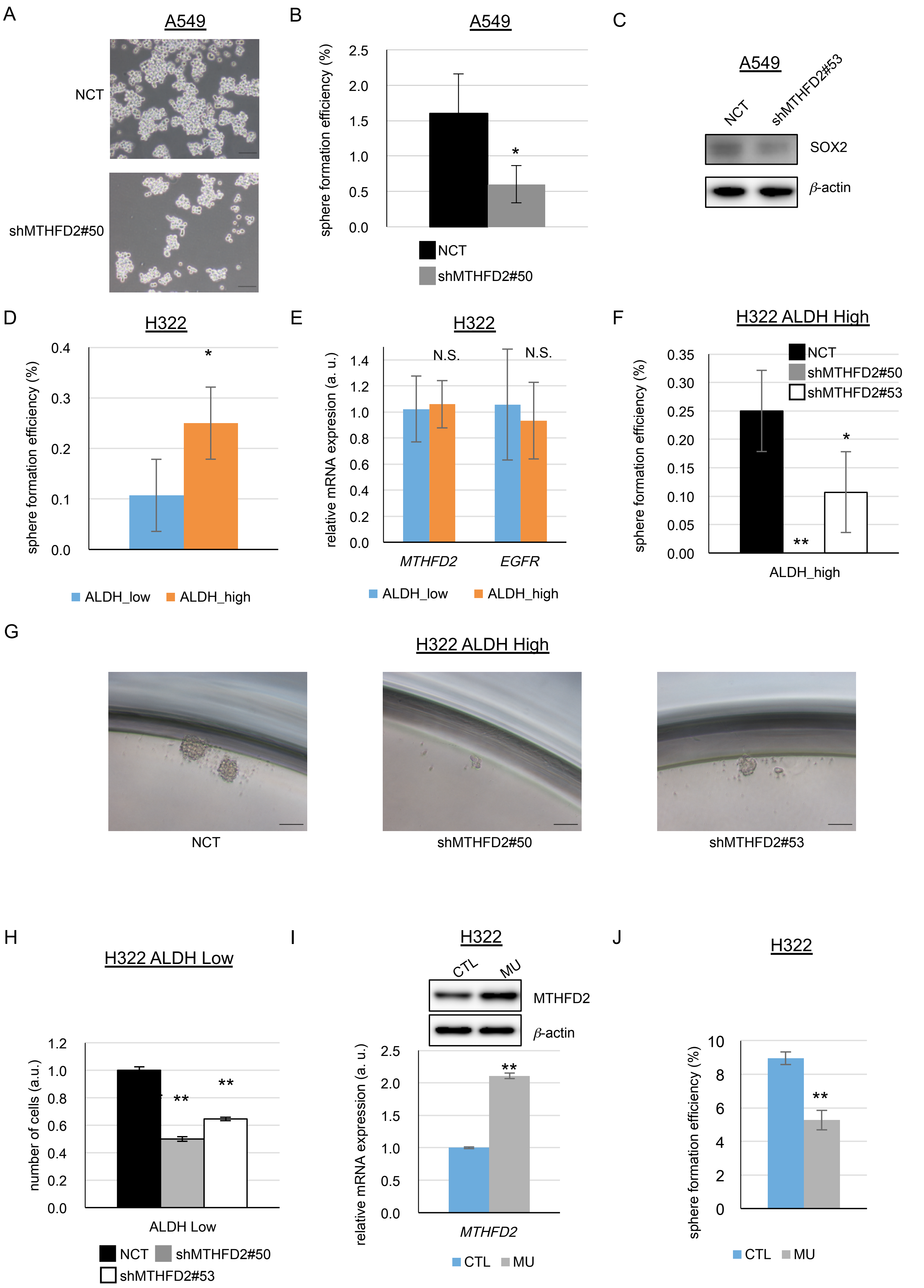

Supplementary Figure 5

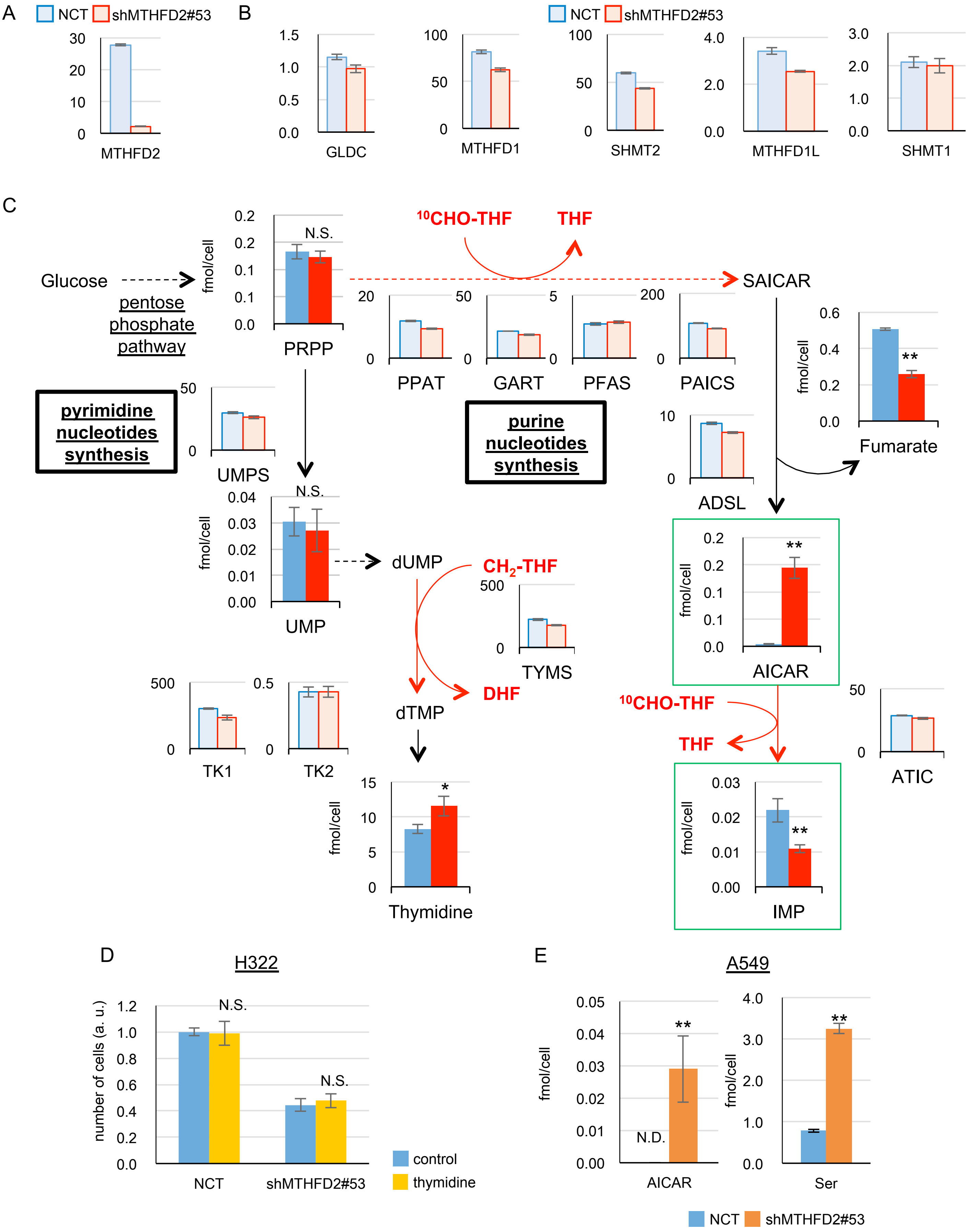

Supplementary Figure 6

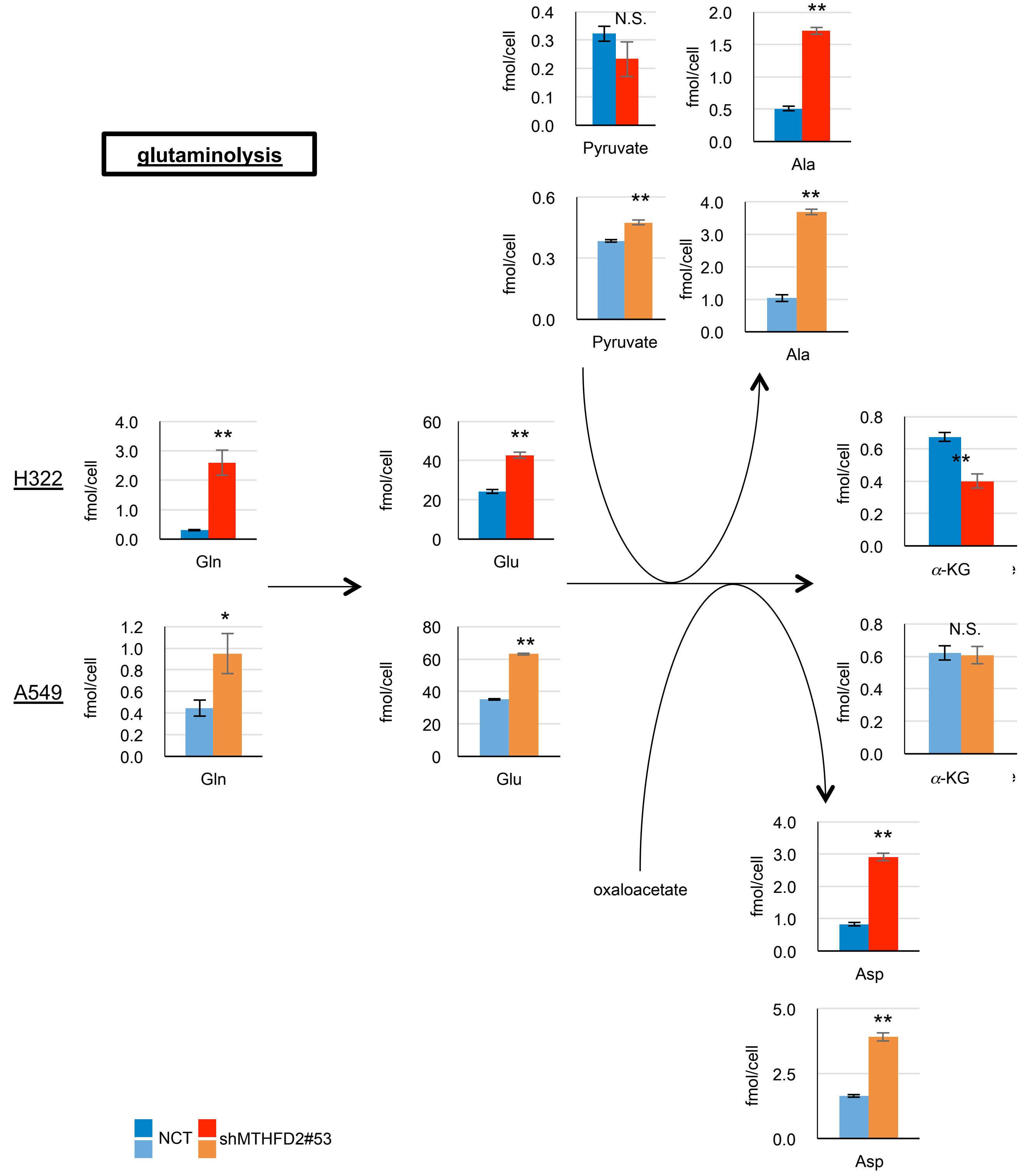

Supplementary Figure 7

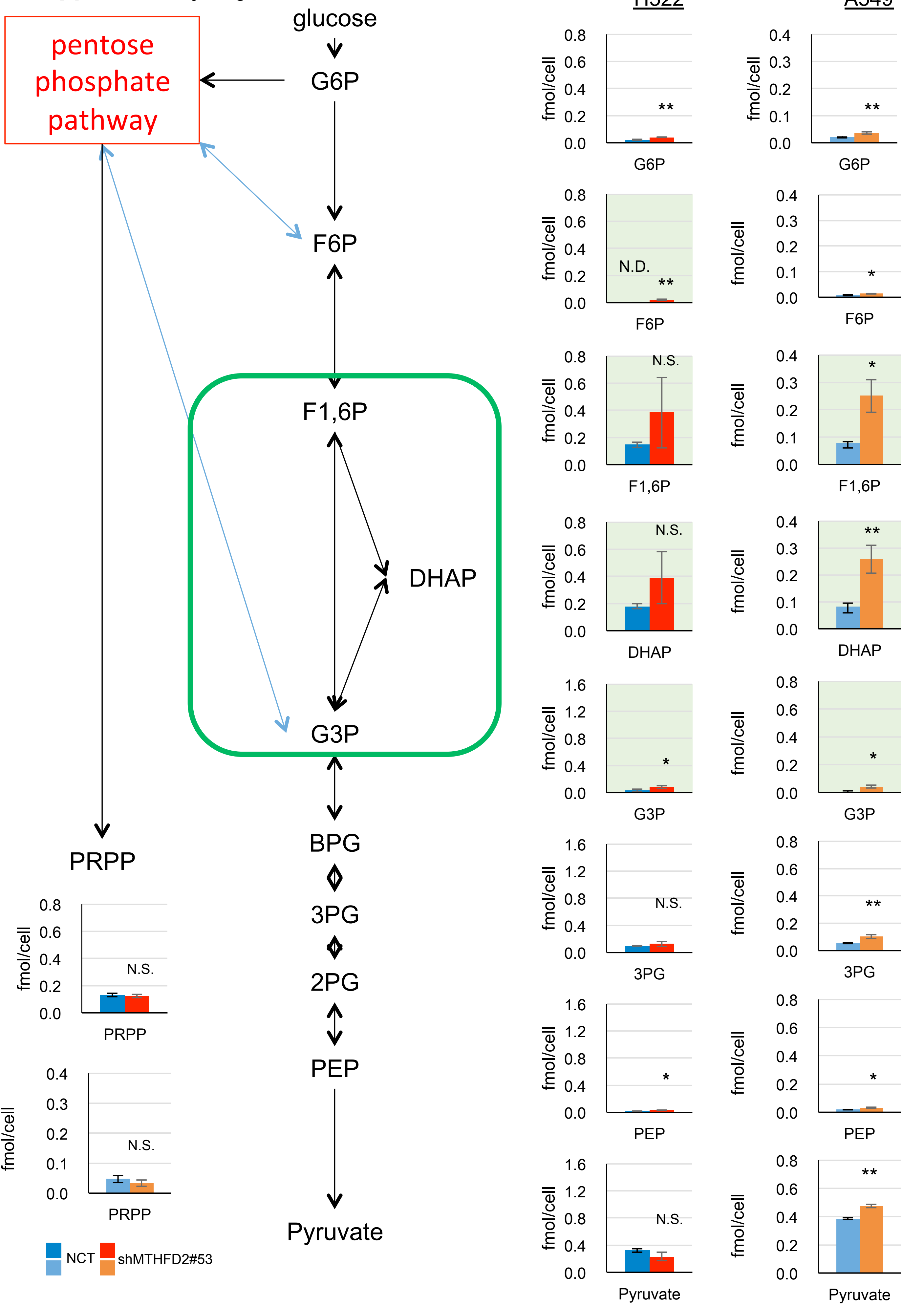

Supplementary Figure 8

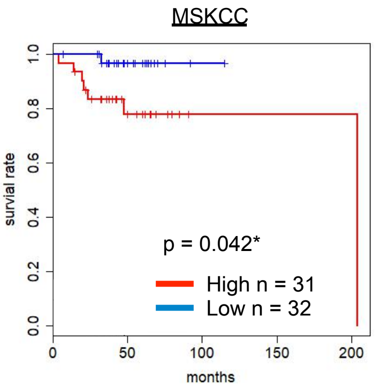

Supplementary Figure 9

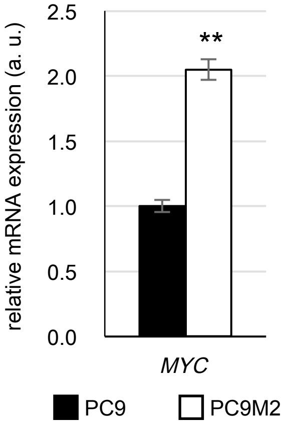

Supplementary Figure 10

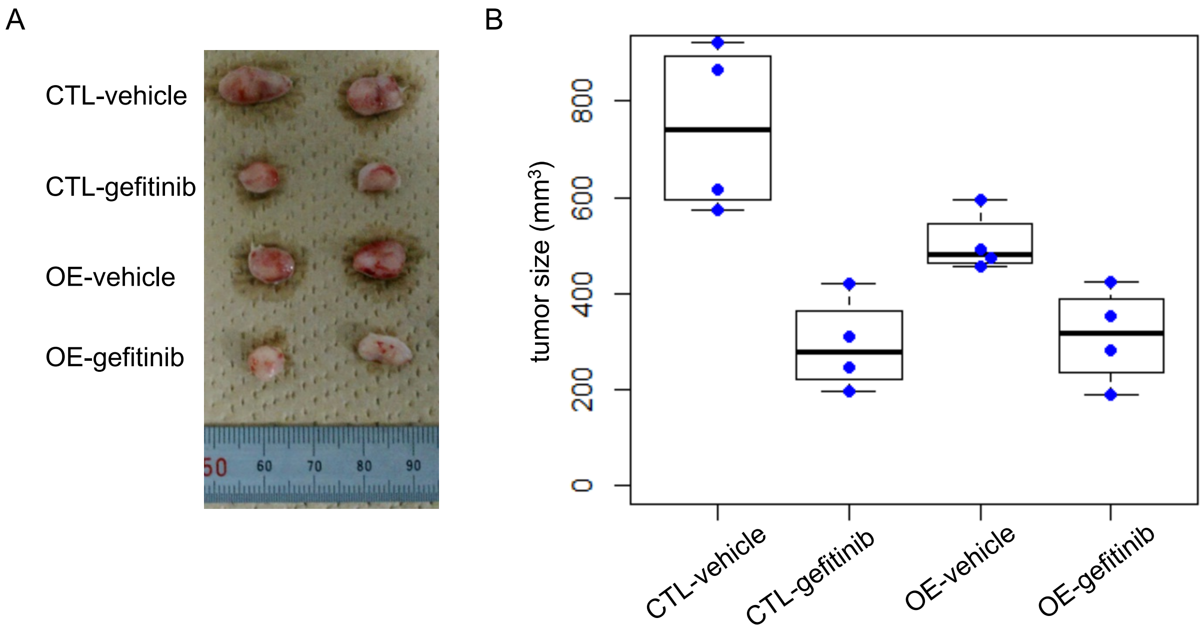

Supplementary Table 1

Hazard ratios for overall survival (cox proportional hazard model)

Data set	Variable	Hazard ratio	95% confidence Interval	p value
NCC	Age	1.0	0.98-1.1	0.27
(n = 195)	Stage (II/I)	3.2	1.6-6.4	0.00080**
	MTHFD2 (High/Low)	2.2	1.1-4.8	0.037*
MSKCC	Age	0.95	0.89-1.0	0.21
(n = 63)	Stage (II/I)	1.9	0.18-20	0.59
	Stage (III/I)	12	1.9-74	0.0076**
	MTHFD2 (High/Low)	14	1.4-140	0.025**

Supplementary Table 2

Dependence on MTHFD2 for cell growth

Cell line	Mutation status of *EGFR*	Gefitinib sensitivity	MTHFD2 dependency
HCC827	Five amino acids deletion in *EGFR*	sensitive	-0.973
HCC827GR5	Five amino acids deletion in *EGFR* and *MET* amplification	resistant	-2.37
NCIH1975	Mutations L858R and T790M in *EGFR*	resistant	-2.88

Database analysis was carried out by using Project Achilles, https://portals.broadinstitute.org/achilles. A large negative value means cell growth activity is strongly dependent on MTHFD2. HCC827 cells harbor gefitinib-sensitive deletion of 5 amino acids, E746 to A750, in *EGFR*. HCC827GR5 cells are derived from HCC827 cells and show acquired gefitinib-resistance due to *MET* amplification. NCIH1975 cells harbor gefitinib-sensitive mutation, L858R, but show acquired gefitinib-resistance due to secondary mutation, T790M, in *EGFR*.
