# Supplementary material for: Cancer stem-like properties and gefitinib resistance are dependent on purine synthetic metabolism mediated by the mitochondrial enzyme MTHFD2
Source: Oncogene. 2018 Dec 7;38(14):2464–81. doi: 10.1038/s41388-018-0589-1 (PMC6484769; doi:10.1038/s41388-018-0589-1)
Supplement: Supplementary file 2 — Figure legends for Supplementary figures clean [file 41388_2018_589_MOESM2_ESM.pdf]

FIGURE LEGENDS FOR SUPPLEMENTARY FIGURES

Supplementary Figure 1. MTHFD2 is a druggable target against cancer.

(A) Kaplan-Meier survival curves of patients in the NCC cohort. Red and blue lines depict survival curves of patients with cancer tissues with high and low levels of *MTHFD2* mRNA, respectively. The cut-off value was determined as the median value of the expression levels of *MTHFD2* mRNA. P-values were calculated with a log-rank test. (B) Comparison of *MTHFD2* mRNA expression levels between normal lung and lung cancer tissues obtained from the Oncomine database. The upper and lower dots represent maximum and minimum values, respectively. The upper and lower ends of the whiskers represent the 90th and 10th percentile values, and the sides of the boxes represent the 75th and 25th percentile values. The lines in the boxes represent median values. (C) Modeled structure of human MTHFD2 (green) with its druggable binding sites (yellow, cyan, flesh, white, purple, and orange) detected by PockDrug-Server. P2 with p4, and p5 are putative NAD(H) and THF derivative binding sites, respectively. (D) The 180 degree-rotated structure of the modeled structure depicted in (C).

Supplementary Figure 2. Expression of MTHFD2 in lung cancer cell lines.

(A) Western blot for MTHFD2 with or without EGF stimulation (100 ng/ml) for 8 hrs. β-actin was used as loading control. Intensities of normalized value of MTHFD2 are depicted. (B) Immunocytochemistry by using anti-OXCT (mitochondria marker) antibody, anti-MTHFD2 antibody and DAPI. Scale bar is 10 μm.

Supplementary Figure 3. Reduced cellular growth following knockdown of *MTHFD2*.

(A) Western blot for MTHFD2 in A549 cells in which *MTHFD2* was knocked down with two different shRNAs. NCT, negative control; shMTHFD2#50 and shMTHFD2#53, *MTHFD2*

knockdown. (B) Cell proliferation assay of A549 cells *in vitro*. Experiments were performed three times and the representative results were presented. The data are represented as mean$\pm$SD, N = 3. (C) Cell cycle analysis of *MTHFD2*-knockdown H322 cells. (D) Tumor growth assay of A549 cells *in vivo*. The data are represented as mean$\pm$SEM, N = 6. (E) Immunohistochemistry for expression of MTHFD2 in tumors derived from the H322 xenograft model (upper panel). Scale bar, 100 μm. Western blot for MTHFD2 in tumors derived from the H322 xenograft model (lower panel). No. 1-5 indicates lysate from each tumor. (F) Immunohistochemistry for Ki-67 in tumors derived from the H322 xenograft model. Scale bar, 100 μm (left panel). Quantitation of number of Ki-67-positive cells. The data are represented as mean$\pm$SD (right panel). (G) Western blot for MTHFD2 in SAECs in which *MTHFD2* was knocked-downed with two different shRNAs. (H) Cell proliferation assay of SAECs *in vitro*. Experiments were performed three times and the representative results were presented. The data are represented as mean$\pm$SD, N = 4. Statistical significance is calculated with a two-tailed unpaired t-test; N.S. not significant, *$p < 0.05$, **$p < 0.01$.

Supplementary Figure 4. Reduced cellular growth and cancer stem-like properties following knockdown of *MTHFD2*.

(A) Bright field images of tumor spheroids of *MTHFD2*-knockdown A549 cells. Scale bar, 100 μm. (B) Sphere formation efficiency of *MTHFD2*-knockdown A549 cells. Experiments were performed three times and the representative results were presented. The data are represented as mean$\pm$SD, N = 4. (C) Western blot for SOX2 in *MTHFD2*-knockdown A549 cells. (D) Sphere formation efficiency of $ALDH^{high}$ or $ALDH^{low}$ population of H322 cells. Experiments were performed three times and the representative results were presented. The data are represented as mean$\pm$SD, N = 4. (E) mRNA expression of *MTHFD2* and *EGFR* in $ALDH^{high}$ or $ALDH^{low}$

population of H322 cells measured by quantitative RT-PCR. The data are represented as mean$\pm$SD, N = 3. (F) Sphere formation efficiency of *MTHFD2*-knockdown ALDH^{high} population of H322 cells. Experiments were performed three times and the representative results were presented. The data are represented as mean$\pm$SD, N = 4. (G) Bright field images of tumor spheroids of *MTHFD2*-knockdown ALDH^{high} population of H322 cells. Scale bar, 100 μm. (H) Cell proliferation assay of ALDH^{low} population of H322 cells *in vitro*. Experiments were performed three times and the representative results were presented. The data are represented as mean$\pm$SD, N = 4. (I) Western blot for MTHFD2 in control vector-transduced cells (CTL) and cells overexpressing mutant MTHFD2 (MU). *β*-actin was used as loading control (upper panel). mRNA expression of *MTHFD2* in CTL and MU cells measured by quantitative RT-PCR (lower panel). (J) Sphere formation efficiency of CTL and MU cells. Experiments were performed three times and the representative results were presented. The data are represented as mean$\pm$SD, N = 4. Statistical significance is calculated with a two-tailed unpaired t-test; N.S. not significant, *p < 0.05, **p < 0.01.

Supplementary Figure 5. Knockdown of MTHFD2 did not significantly alter expression levels of one-carbon metabolism related-major enzymes and purine and pyrimidine nucleotide synthesis enzymes.

mRNA levels of *MTHFD2* (A) and major enzymes (B) in folate-mediated 1C metabolism: glycine decarboxylase (GLDC), MTHFD1, serine hydroxymethyltransferase (SHMT)-2, MTHFD1L, and SHMT-1. (C) mRNA levels of major enzymes in purine and pyrimidine nucleotide synthesis pathways. : phosphoribosylpyrophosphate amidotransferase (PPAT), phosphoribosylglycinamide formyltransferase (GART), phosphoribosylformylglycinamidine synthase (PFAS), phosphoribosylaminoimidazole carboxylase (PAICS), adenylosuccinate lyase

(ADSL), and 5-aminoimidazole-4-carboxamide ribonucleotide formyltransferase/inositol monophosphate (IMP) cyclohydrolase (ATIC). (D) Cell proliferation assay in the presence of thymidine. Experiments were performed three times and the representative results were presented. The data are represented as mean±SD, N = 3. (E) Concentrations of AICAR and Ser in *MTHFD2* knockdown A549 cells. NCT, negative control; shMTHFD2#53, *MTHFD2* knockdown. The data are represented as mean±SD, N = 3. Statistical significance is calculated with a two-tailed unpaired t-test; N.S., not significant, *p < 0.05, **p < 0.01.

Supplementary Figure 6. Concentrations of metabolites in glutaminolysis under *MTHFD2*-knockdown.

The data are represented as mean±SD, N = 3. Statistical significance is calculated with a two-tailed unpaired t-test; N.S., not significant, **p < 0.01.

Supplementary Figure 7. Concentrations of metabolites in glycolysis under *MTHFD2*-knockdown.

Concentrations of following metabolites are indicated: glucose 6-phosphate (G6P), fructose 6-phosphate (F6P), fructose-1,6-bisphosphate (F1,6P), dihydroxyacetone phosphate (DHAP), glyceraldehyde 3-phosphate (G3P), glyceraldehyde 3-phosphate (BPG), 3-phospho-D-glycerate (3PG), 2-phospho-D-glycerate (2PG), phosphoenolpyruvate (PEP). F6P and G3P are used in both glycolysis and pentose phosphate pathway indicated by blue arrows. Concentrations of F1,6P, DHAP and G3P (in a green circle) in *MTHFD2*-knockdown H322 and A549 cells were more than two fold higher than those of control cells. The graphs of these three metabolites are colored in light green. One mole of F1,6P is catalized into one mole of DHAP and G3P. Then DHAP is converted to one mole of G3P. Thus G3P is catalized into 2 fold more amount of

F1,6P. To easily compare the concentrations of each metabolite, the scale of the vertical axes of the graphs of concentrations of G3P and its downstream metabolites are two fold larger than those of F1,6P, DHAP and their upstream metabolites. The data are represented as mean$\pm$SD, N = 3. Statistical significance is calculated with a two-tailed unpaired t-test; N.S., not significant, *$p < 0.05$, **$p < 0.01$.

Supplementary Figure 8. Kaplan-Meier survival curves of patients in the MSKCC cohort.

Red and blue lines depict survival curves of patients with cancer tissues with high and low levels of *MTHFD2* mRNA, respectively. The cut-off value was determined as the median value of the expression levels of *MTHFD2* mRNA. P-values were calculated with a log-rank test.

Supplementary Figure 9. Increased expression levels of *MYC* in PC9M2 cells.

Expression of *MYC* in PC9 and PC9M2 cells measured by quantitative RT-PCR. The data are represented as mean$\pm$SD, N = 3. Statistical significance is calculated with a two-tailed unpaired t-test; **$p < 0.01$.

Supplementary Figure 10. Overexpression of MTHFD2 confers gefitinib-resistance in PC9 cells.

(A) Tumors of empty vector-transduced control PC9 cells (CTL) or MTHFD2-overexpressing PC9 cells (OE) xenograft model treated with vehicle or gefitinib. (B) Tumor size measurement. Blue circles indicate sizes of each tumor. Whiskers were elongated to the largest and smallest values, which are not outliers. The sides of the boxes represent the 75th and 25th percentile values. The lines in the boxes represent median values. N=4.
